# Encoding of Natural Sounds at Multiple Spectral and Temporal Resolutions in the Human Auditory Cortex

**DOI:** 10.1371/journal.pcbi.1003412

**Published:** 2014-01-02

**Authors:** Roberta Santoro, Michelle Moerel, Federico De Martino, Rainer Goebel, Kamil Ugurbil, Essa Yacoub, Elia Formisano

**Affiliations:** 1 Department of Cognitive Neuroscience, Faculty of Psychology and Neuroscience, Maastricht University, Maastricht, The Netherlands; 2 Maastricht Brain Imaging Center (MBIC), Maastricht, The Netherlands; 3 Center for Magnetic Resonance Research, Department of Radiology, University of Minnesota, Minneapolis, Minnesota, United States of America; 4 Department of Neuroimaging and Neuromodeling, Netherlands Institute for Neuroscience, Royal Netherlands Academy of Arts and Sciences (KNAW), Amsterdam, The Netherlands; Indiana University, United States of America

## Abstract

Functional neuroimaging research provides detailed observations of the response patterns that natural sounds (e.g. human voices and speech, animal cries, environmental sounds) evoke in the human brain. The computational and representational mechanisms underlying these observations, however, remain largely unknown. Here we combine high spatial resolution (3 and 7 Tesla) functional magnetic resonance imaging (fMRI) with computational modeling to reveal *how* natural sounds are represented in the human brain. We compare competing models of sound representations and select the model that most accurately predicts fMRI response patterns to natural sounds. Our results show that the cortical encoding of natural sounds entails the formation of multiple representations of sound spectrograms with different degrees of spectral and temporal resolution. The cortex derives these multi-resolution representations through frequency-specific neural processing channels and through the combined analysis of the spectral and temporal modulations in the spectrogram. Furthermore, our findings suggest that a spectral-temporal resolution trade-off may govern the modulation tuning of neuronal populations throughout the auditory cortex. Specifically, our fMRI results suggest that neuronal populations in posterior/dorsal auditory regions preferably encode coarse spectral information with high temporal precision. Vice-versa, neuronal populations in anterior/ventral auditory regions preferably encode fine-grained spectral information with low temporal precision. We propose that such a multi-resolution analysis may be crucially relevant for flexible and behaviorally-relevant sound processing and may constitute one of the computational underpinnings of functional specialization in auditory cortex.

## Author Summary

How does the human brain analyze natural sounds? Previous functional neuroimaging research could only describe the response patterns that sounds evoke in the human brain at the level of preferential regional activations. A comprehensive account of the neural basis of human hearing, however, requires deriving computational models that are able to provide quantitative predictions of brain responses to natural sounds. Here, we make a significant step in this direction by combining functional magnetic resonance imaging (fMRI) with computational modeling. We compare competing computational models of sound representations and select the model that most accurately predicts the measured fMRI response patterns. The computational models describe the processing of three relevant properties of natural sounds: frequency, temporal modulations and spectral modulations. We find that a model that represents spectral and temporal modulations jointly and in a frequency-dependent fashion provides the best account of fMRI responses and that the functional specialization of auditory cortical fields can be partially accounted for by their modulation tuning. Our results provide insights on how natural sounds are encoded in human auditory cortex and our methodological approach constitutes an advance in the way this question can be addressed in future studies.

## Introduction

Understanding how natural sounds and scenes are processed in the human auditory cortex remains a major challenge in auditory neuroscience. Current models of auditory cortical processing describe the sound-evoked neural response patterns at the level of preferential regional activations for certain behavioral tasks (e.g. localization vs recognition [1], [2]), sound categories (e.g. voices, speech [3]) and (complex) acoustic features [4], [5]. However, the computational and representational mechanisms underlying these responses remain largely unknown. The overall aim of the present study is to derive a computational model of *how* natural sounds are encoded in the human brain by combining high-resolution fMRI (3 and 7 Tesla) with computational modelling.

**Figure 1:**
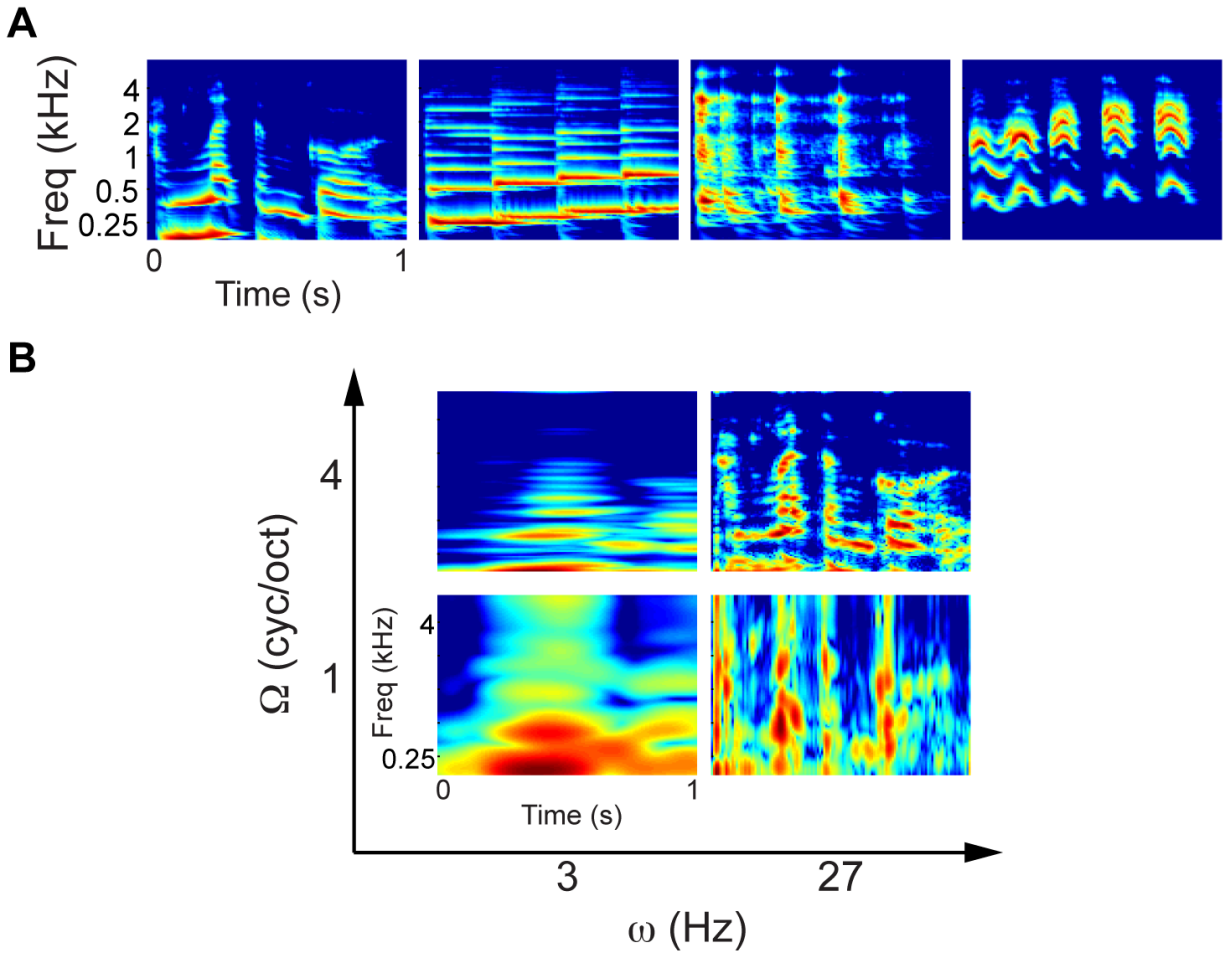
Sound examples and multi-resolution decomposition. (A) Spectrogram of four exemplary natural sounds used in this study as extracted by the computational model mimicking early auditory processing. Natural sounds exhibit modulations of acoustic energy along both frequency and time. (B) Multi-resolution representation of the leftmost spectrogram of panel A. Different “views” are obtained as output of modulation channels tuned to specific spectral modulation (Ω) and temporal modulation (ω) frequencies. Each channel represents the spectrogram with a different combination of spectral and temporal detail.

Most natural sounds are characterized by modulations of acoustic energy in both the spectral and temporal dimensions (Figure 1A). These modulations occur at multiple scales [6] and are crucial for behaviorally relevant auditory processing such as speech intelligibility [7]–[10]. Psychophysical investigations indicate that humans are able to detect and discriminate modulations that occur in one dimension alone (temporal: [11]; spectral: [12]) as well as combined spectro-temporal modulations [9]. Similarly, neurophysiological studies in animals and humans have revealed neuronal tuning for temporal modulations [13]–[15] and spectral modulations [16] alone, and the combination of the two [17]–[21]. This evidence suggests that spectral and temporal modulations are critical stimulus dimensions for the processing of sounds in the auditory cortex. Just as the cochlea generates multiple “views” of the sound pressure wave at different frequencies, an explicit encoding of spectral and temporal modulations would allow the cortex generating multiple “views” of the sound spectrogram with different degrees of spectral and temporal resolution [22] (Figure 1B). Multiple simultaneous representations of the same incoming sounds may be crucially relevant for enabling flexible behavior, as different goal-oriented sound processing (e.g. sound localization or identification) may benefit from different types of representations. Furthermore, the representations of sounds at multiple resolutions may provide the computational basis for binding acoustic elements in sound mixtures and solve complex auditory scenes [23].

Despite extensive investigations in a variety of experimental settings, the specific computational mechanisms used by the human auditory cortex to represent energy modulations in the spectrogram of natural sounds are still a matter of speculation. Here, we use an fMRI “encoding” approach [24] to compare competing computational models of sound representations and select the *best* model as the one that can predict most accurately fMRI response patterns to natural sounds. We focus on three well-defined aspects of the representation of spectral and temporal modulations: (1) *dependency*, (2) *frequency specificity*, and (3) *spatial organization*.

Dependency refers to the relation between spectral and temporal processing. The spectrogram of natural sounds is characterized by concurrent spectral and temporal modulations and these sound qualities might be represented jointly or independently of each other. An *independent* representation implies separate processing mechanisms for spectral and temporal modulations, such that the response to one dimension is invariant to a change in the other dimension. By contrast, a *joint* representation relies on combined selectivity for the conjunction of spectral and temporal modulations. The joint representation can be modeled as an array of spectro-temporal filters that are selective for combinations of spectral and temporal modulations (Figure S1A), whereas the independent representation can be seen as a bank of filters that are selective for either temporal or spectral modulations (Figure S1B). In other words, the two models differ with respect to the dimensions employed by the auditory cortex to encode natural sounds (combined spectro-temporal modulations, and spectral and temporal modulations alone, respectively). Testing for the interdependency of spectral and temporal modulation processing has relevant implications, as the superiority of such a model would indicate that results obtained using sounds that only vary along one dimension (e.g. amplitude modulated tones or stationary ripples) cannot be generalized to mechanisms of representation and processing of natural sounds.

The analysis of the spectro-temporal modulation content of the sound spectrogram can be global (2D Fourier transform) or localized (e.g wavelet transform). A global representation indicates integration along the frequency axis, while in a local analysis spectral and temporal modulations are encoded in a *frequency-specific* fashion. Frequency specific responses are ubiquitous in the auditory cortex; yet it is not clear how this dimension is exploited for the representation of natural sounds. Understanding the nature of the modulation analysis performed by the human auditory cortex can provide insights about the functional role of this representational mechanism.

Finally, the third aspect that we consider is the existence and layout of a large-scale spatial organization of spectro-temporal modulation tuning. Topographic maps of stimulus dimensions are a well-established organizational principle of the auditory cortex [25]. In humans, the primary [26] as well as the non-primary [27] auditory cortex contain multiple topographic representations of sound frequency (tonotopic maps). Beyond tonotopy, however, the spatial organization of other sound features remains elusive [25]. Our methodological approach provides the possibility to obtain maps of multiple sound features and feature-combinations from the same set of fMRI responses and within the ecologically and behaviorally-relevant context of natural sounds processing. Here, we exploit this possibility to study the regional specificity and the spatial organization of spectro-temporal modulation tuning. Such knowledge can reveal the representational and computational basis underlying the functional specialization of auditory cortical subdivisions.

Our results show that the human brain forms multiple representations of incoming natural sounds at distinct spectral and temporal resolutions. The encoding of spectral and temporal modulations is *joint* and *frequency-specific* and is governed by a trade-off between spectral and temporal resolution. Regional variations of voxels modulation preference put forward the hypothesis that the functional specialization of auditory cortical fields can be partially accounted for by their modulation tuning.

## Results

We modeled the data from two fMRI experiments in humans (3 [27] and 7T [28], [29]). In both experiments, fMRI responses were recorded from the auditory cortex while subjects (n = 5, different for the two experiments) listened to a large set of natural sounds, including speech samples, music pieces, animal cries, scenes from nature, and tool sounds (see Materials and Methods and Text S1).

### Prediction accuracy of the joint frequency-specific MTF-based model

**Figure 2:**
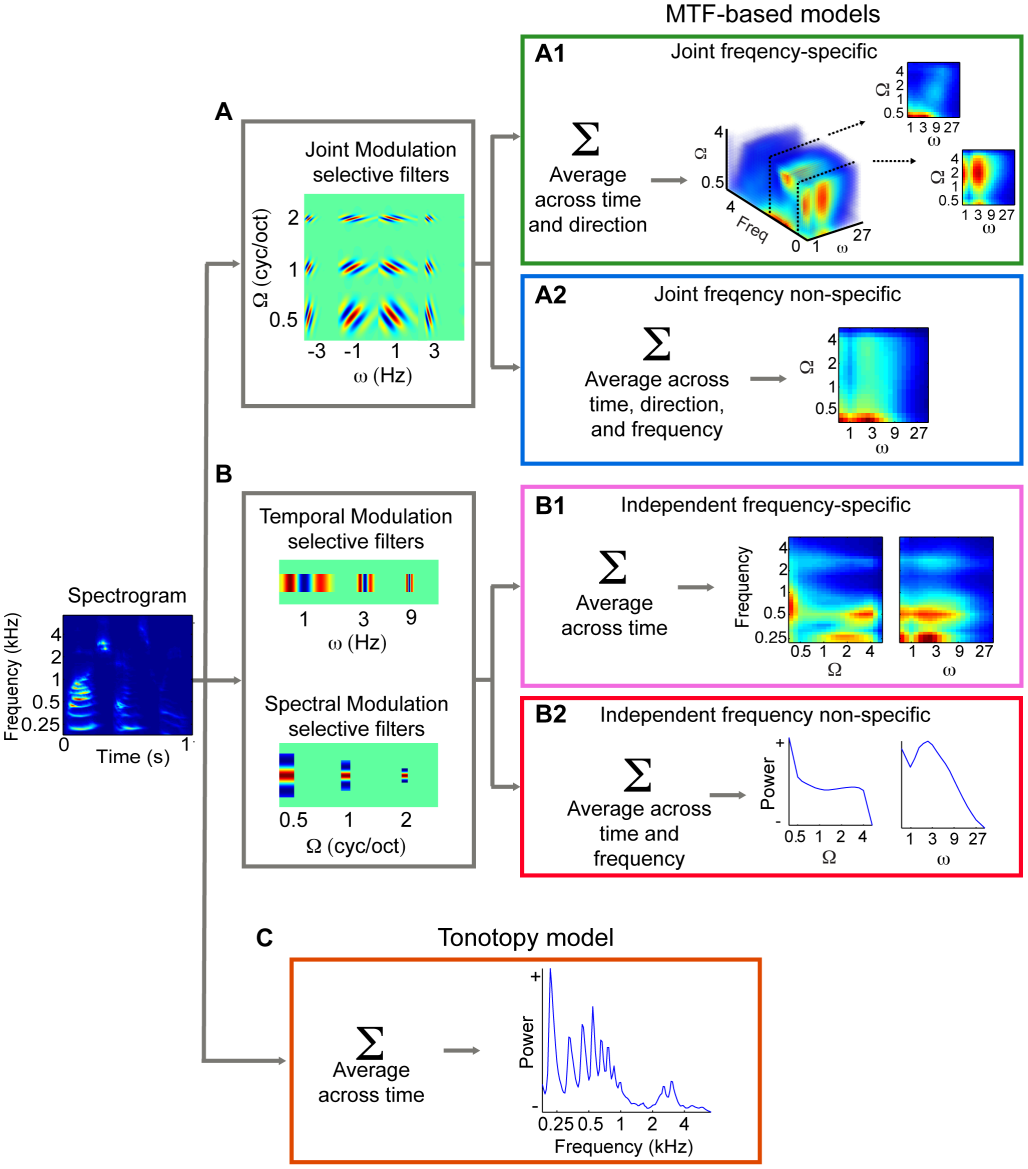
Overview of candidate encoding models. (A) Joint modulation selective filters. (A1) Joint frequency specific: the spectrogram is filtered with a bank of modulation selective filters at different spectral modulations (Ω), temporal modulations (ω), and direction (upwards/downwards). The output of the filter bank is averaged across time and direction to yield a reduced representation of modulation energy as a function of Ω, ω, and frequency. The joint frequency-specific MTF-based model predicts that fMRI responses vary linearly with this representation, i.e. sounds that differ with respect to any of the three dimensions will elicit different responses. (A2) Joint frequency non-specific: the 3D modulation representation is averaged across frequency to yield a global measure of modulation energy. By concatenating modulation and frequency content (not shown here, see tonotopy model), the joint frequency non-specific model predicts separate processing for global, joint modulations and frequency. (B) Independent modulation selective filters. (B1) Independent frequency-specific: the spectrogram is filtered with purely spectral and purely temporal modulation selective filters and the output is averaged over time. This yields separate representations of spectral and temporal modulation energy as a function of frequency. The independent frequency-specific model predicts that the response of a voxel dedicated to spectral (temporal) processing will not be affected by a change in temporal (spectral) modulation content. (B2) Independent frequency non-specific: the two separate representations of spectral and temporal modulation energy are averaged across frequency to yield the global spectral and temporal modulation content. This representation is concatenated with the frequency content (not shown here, see tonotopy model) to simulate separate processing for frequency, spectral and temporal modulations. (C) Tonotopy model: the spectrogram is averaged over time and voxels are modeled as frequency selective units, whose response varies linearly with the frequency content of the input stimuli.

**Figure 3:**
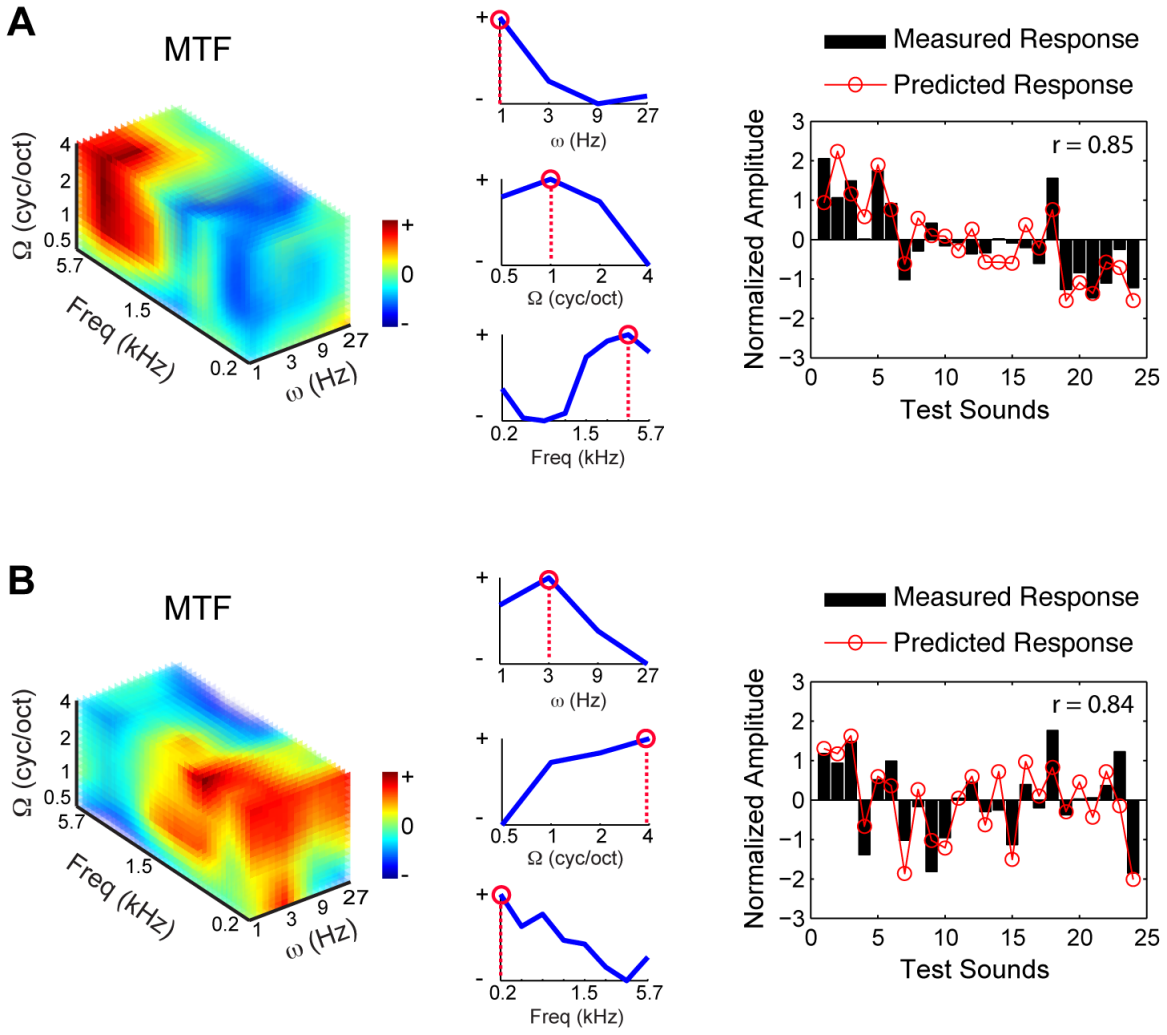
Sample MTFs, data and model prediction for two exemplary voxels (subject S10). (A–B) Left: MTFs as estimated by the joint frequency-specific MTF-based model. The color code indicates the voxel's sensitivity to a given combination of frequency, spectral and temporal modulation. MTFs have been interpolated for display purposes. Middle: Marginal response profiles for temporal modulation (top), spectral modulation (middle) and frequency (bottom). Red circles and dashed lines indicate voxels' characteristic spectral modulation, temporal modulation and frequency, computed as the point of maximum of the marginal profiles (see Materials and Methods). Right: Measured and predicted response to the 24 stimuli in the test set. Responses are shown in z-score units. r indicates Pearson's correlation coefficient.

We applied an “encoding” approach (see [24] and Figure S2) and compared several computational models of auditory processing. A first model we tested describes auditory cortical neurons as a bank of frequency-localized filters with joint selectivity for spectral and temporal modulations (see [22] and Materials and Methods). Considering that one voxel reflects the mass activity of a great number of neurons, we modelled each voxel's receptive field as a combination of modulation selective filters, each tuned to a different spectral modulation, temporal modulation and frequency (Figure 2, panel A). Using a subset of fMRI data (training), we estimated a modulation transfer function (MTF, Figure 2, panel A1) for each voxel (see Figure 3 for two MTF examples). We then assessed the ability of this MTF-based model to accurately predict the fMRI responses in new, independent data sets (testing). In the 3T experiment, training and testing data involved a single set of natural sounds, whereas two completely distinct sound sets were used for the 7T training and testing datasets. We quantified model's prediction accuracy by performing a sound identification analysis [24]. Namely, we used the fMRI activity patterns predicted by the estimated models to identify which sound had been heard among all sounds in the test set. Each testing sound was assigned with a score ranging between 0 and 1 and indicating the rank of the correlation between sound's predicted and measured activity patterns (0 indicates that the predicted activity pattern for a given stimulus was least similar to the measured one among all test stimuli; 1 indicates correct identification). The overall model's accuracy was obtained as the average score across all test sounds (see Materials and Methods).

**Figure 4:**
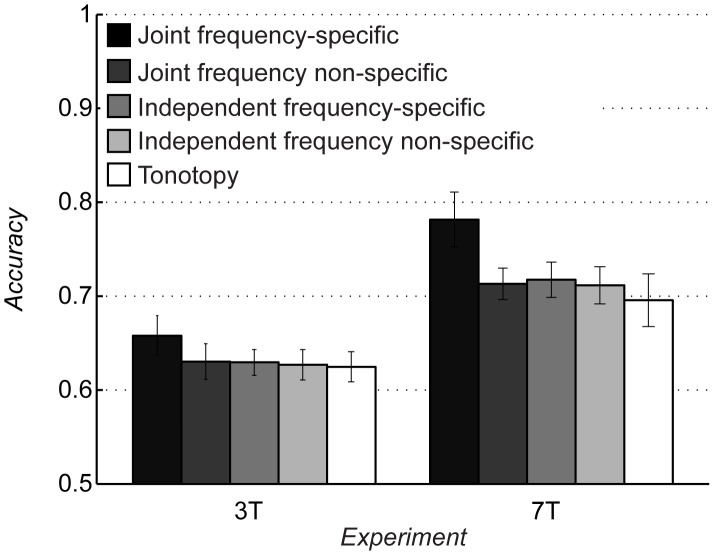
Comparison between models. Bars indicate the prediction accuracy (mean ± SEM, N = 5) for the five models in both the 3T and 7T experiments. The joint frequency-specific MTF-based model showed significantly better prediction accuracy than all other models (see main text). Accuracies are normalized between 0 and 1. Chance level is 0.5.

**Figure 5:**
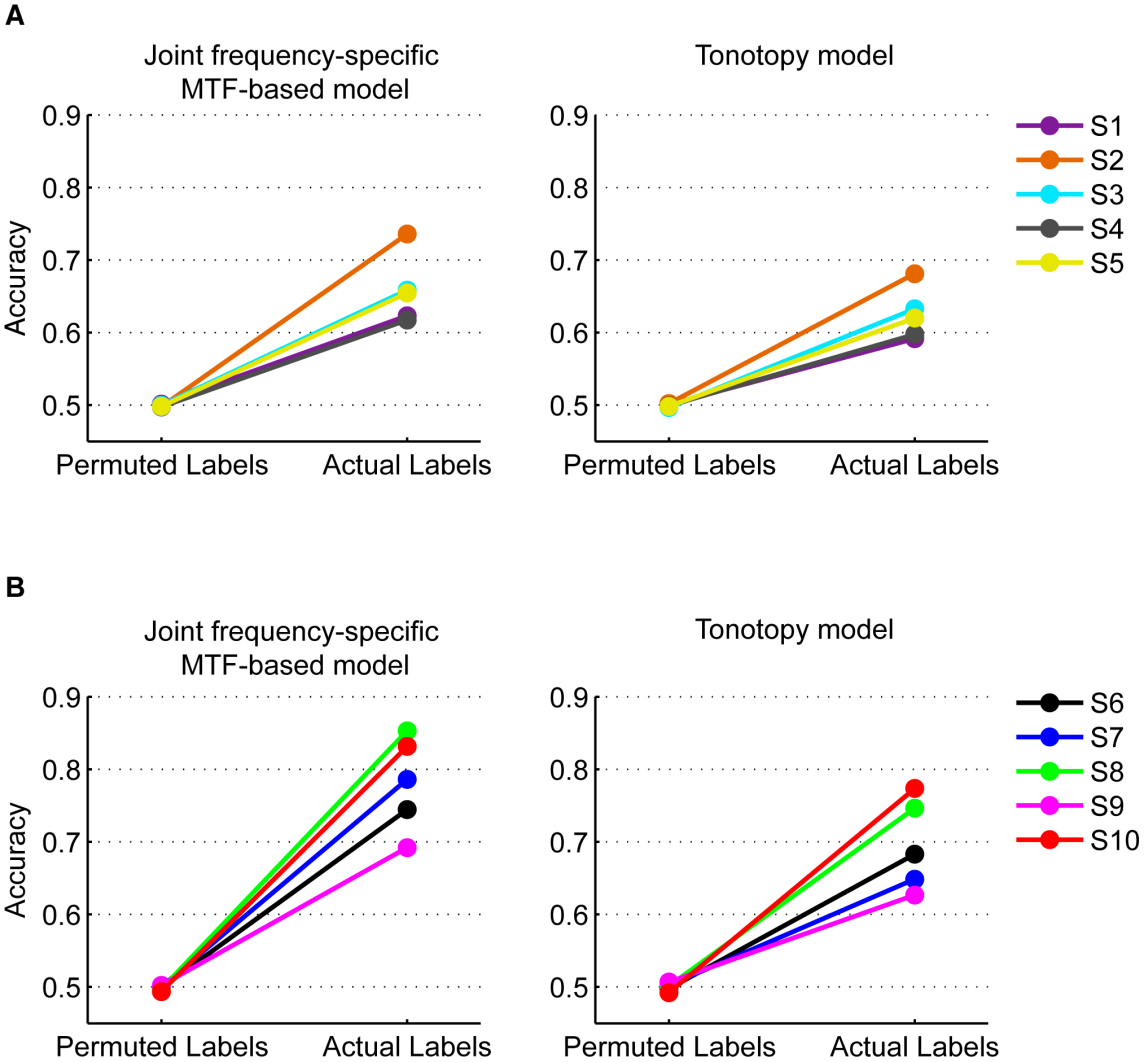
Prediction accuracies for individual participants. Accuracies of the joint frequency-specific MTF-based (left) and tonotopy (right) models are reported for the 3T (A) and 7T (B) datasets. Each panel shows the accuracy obtained with correct labels and the accuracy derived by permuting the sound labels before training the model.

For both the 3T and 7T datasets, the accuracy of the joint frequency-specific MTF-based model was significantly higher than chance (0.5) both at group level (3T: mean [SE] = 0.66 [0.02], p = 0.003; 7T: mean [SE] = 0.78 [0.03], p = 0.002; two-tailed paired t-test; Figure 4) and for each individual subject (p = 0.01 for subject S4, p = 0.005 for all other subjects, permutation test; Figure 5). Remarkably, for the 7T dataset the joint frequency-specific MTF-based model was able to generalize to stimuli not used for parameter estimation.

### Comparison between joint frequency-specific MTF-based model and tonotopy model

FMRI activity from voxels in primary and non-primary auditory regions reflects the tonotopic organization of neural responses. Therefore, as a control analysis we compared the prediction accuracy of the MTF-based model against the prediction accuracy of a tonotopy model, which incorporates the hypothesis that voxels simply reflect information about the frequency content of the stimuli (see Materials and Methods and Figure 2, panel C). The tonotopy model performed above chance both at group level (3T: mean [SE] = 0.62 [0.02], p = 0.002; 7T: mean [SE] = 0.69 [0.03], p = 0.004; two-tailed paired t-test; Figure 4) and for each individual subject (p = 0.015 for subject S4, p = 0.005 for all other subjects, permutation test; Figure 5). However, the tonotopy model performed significantly worse than the joint frequency-specific MTF-based model (3T: p = 0.009; 7T: p = 0.007; two-tailed paired t-test). The significant improvement of the MTF-based over the tonotopy model indicates that a model accounting for the joint, frequency-specific modulation content of the spectrogram is a better representation of fMRI responses to natural sounds.

### Comparison between frequency-specific and non-specific joint MTF-based models

To assess the relevance of frequency-localization in the encoding of joint spectro-temporal modulations, we trained a model that represents frequency and joint modulation content independently of each other (see Materials and Methods and Figure 2, panel A2). The joint frequency non-specific MTF-based model performed above chance both at group level (3T: mean [SE] = 0.63 [0.02], p = 0.004; 7T: mean [SE] = 0.71 [0.02], p = 0.0003; two-tailed paired t-test; Figure 4) and for each individual subject (p = 0.02 for subject S4, p = 0.01 for subject S6, p = 0.005 for all other subjects, permutation test). However, the frequency non-specific model performed significantly worse than the frequency-specific MTF-based model (3T: p = 0.002; 7T: p = 0.021; two-tailed paired t-test).

### Comparison between joint and independent frequency-specific MTF-based models

In order to quantify the contribution of joint selectivity to identification performance, we trained an independent frequency-specific MTF-based encoding model. We modelled each voxel's receptive field as a combination of purely temporal and purely spectral modulation selective filters, operating in a frequency-specific fashion (see Materials and Methods and Figure 2, panels B and B1). The independent model performed above chance both at group level (3T: mean [SE] = 0.63 [0.01], p = 0.001; 7T: mean [SE] = 0.72 [0.02], p = 0.0007; two-tailed paired t-test; Figure 4) and for each individual subject (p = 0.015 for subject S4, p = 0.01 for subject S7, p = 0.005 for all other subjects, permutation test). However, the independent model performed significantly worse than the joint MTF-based model (3T: p = 0.012; 7T: p = 0.011; two-tailed paired t-test).

### Comparison between joint frequency-specific and independent frequency non-specific MTF-based models

As an additional control, we tested a model that simulates independent selectivity for spectral modulations, temporal modulations and frequency (see Materials and Methods and Figure 2, panel B2). The independent frequency non-specific model performed above chance both at group level (3T: mean [SE] = 0.63 [0.02], p = 0.002; 7T: mean [SE] = 0.71 [0.02], p = 0.0008; two-tailed paired t-test; Figure 4) and for each individual subject (p = 0.01 for subject S1, S4 and S9, p = 0.005 for all other subjects, permutation test). However, the independent frequency non-specific model performed significantly worse than the joint frequency-specific MTF-based model (3T: p = 0.011; 7T: p = 0.016; two-tailed paired t-test).

### Spatial distribution of voxels' tuning properties

**Figure 6:**
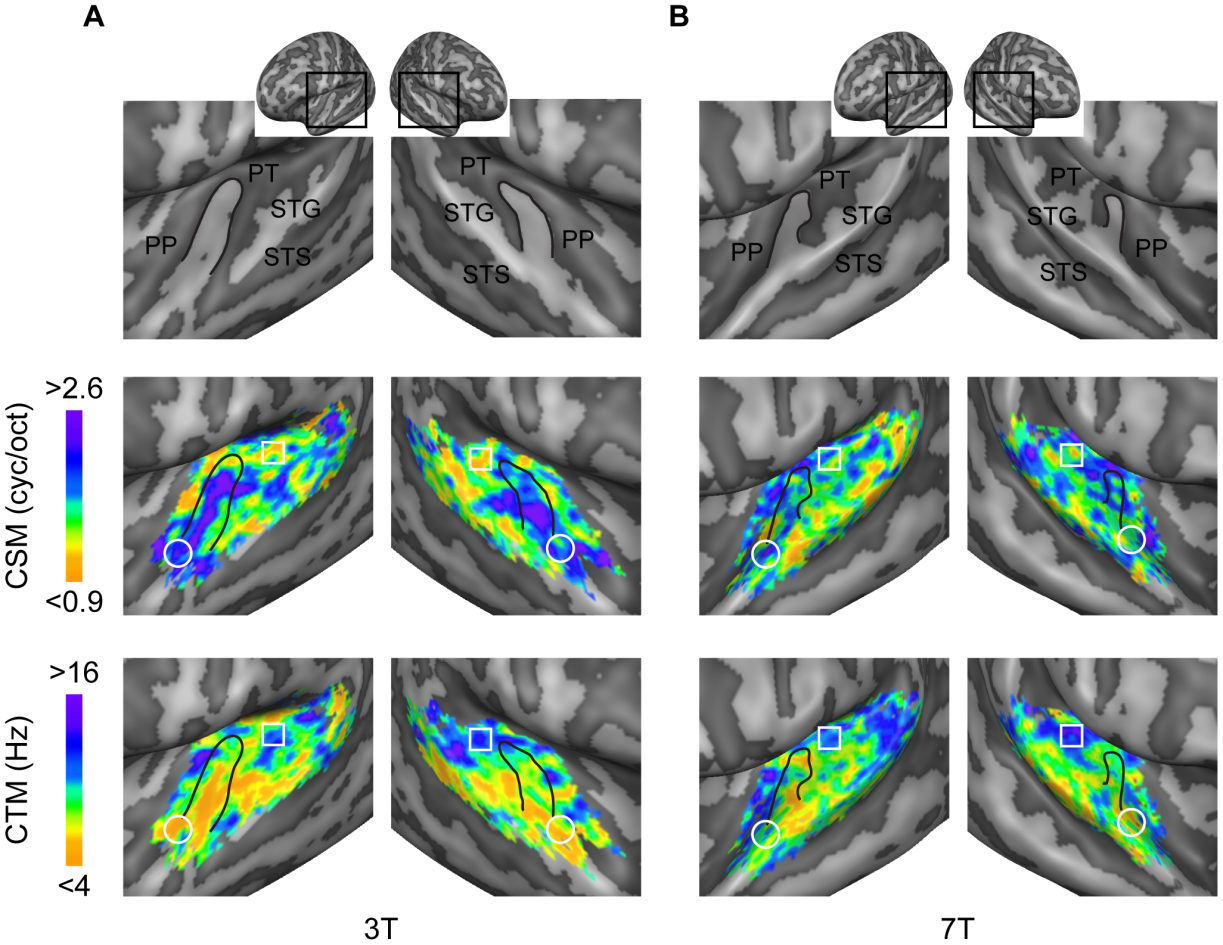
Group maps of CSM and CTM. Maps are displayed for the 3T (A) and 7T (B) datasets. Top: inflated representation of the group cortex. Maps are shown in the cortical region highlighted by the black square. Middle, Bottom: purple denotes tuning for fine (fast) spectral (temporal) structures; orange denotes tuning for coarse (slow) spectral (temporal) features. The white circle and square outline anterior/ventral and posterior/dorsal auditory regions, respectively. The black line indicates HG.

**Figure 7:**
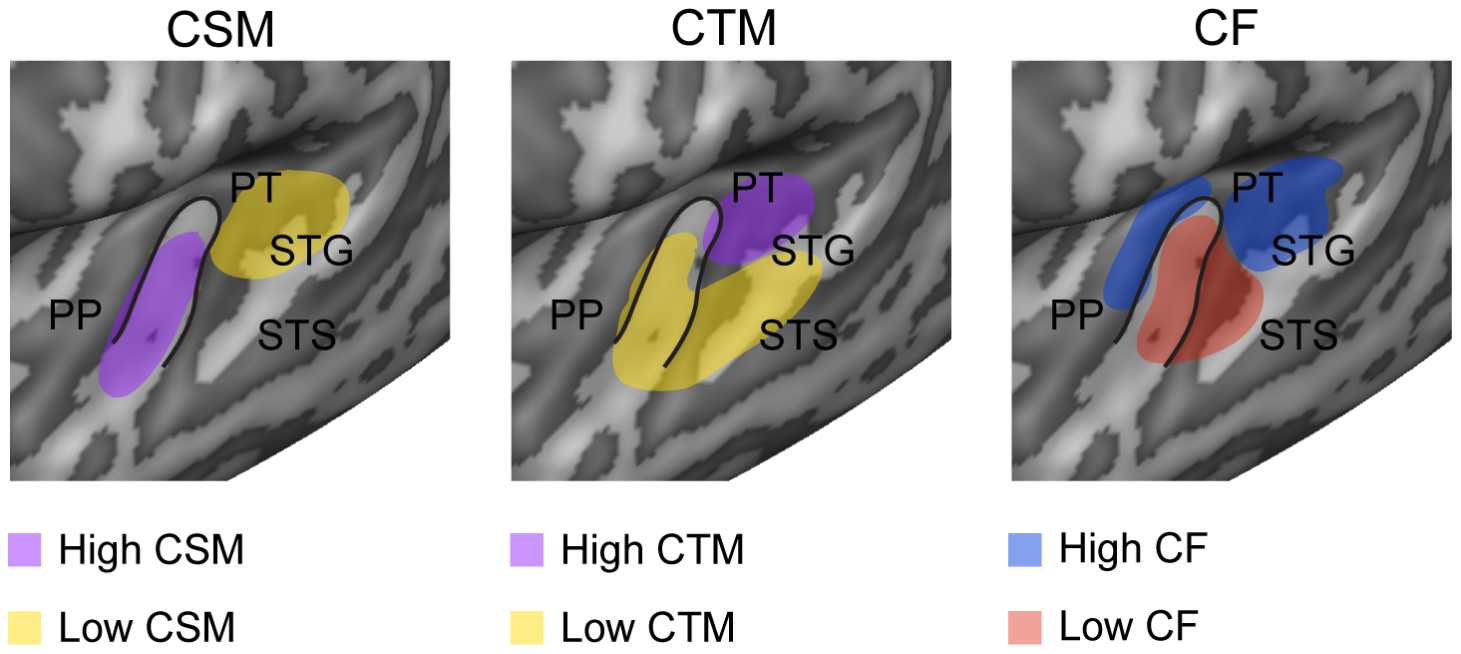
Summary of spatial distribution of voxels' tuning properties. The cartoon is a schematic representation of regional preferences for spectral modulation (left), temporal modulation (middle), and frequency (right). The schematic of spectral and temporal modulation preference summarizes the most evident characteristics emerging from the complex spatial pattern of CSM and CTM. The cartoon of frequency preference shows the main tonotopic gradient in regions along and surrounding HG. Cartoon clusters are superimposed over the left hemisphere of the group cortex as derived from the experiment at 3T. The black line indicates HG.

To investigate the cortical topography of voxels tuning properties, we computed maps of voxels characteristic spectral modulation (CSM), temporal modulation (CTM) and frequency (CF). For each feature, the estimated MTF was marginalized across irrelevant dimensions (i.e. spectral and temporal modulations for CF) and the point of maximum of the marginal sum was assigned as the voxel's preferred feature value (see example in Figure 3). We obtained maps of CSM, CTM and CF by color-coding the voxels' preferred values and projecting them onto an inflated representation of the subject's cortex (see Materials and Methods). Maps of CF confirmed the presence of multiple tonotopic gradients in primary auditory regions (Heschl's gyrus - HG) and surrounding superior temporal cortex [27] (Figure S3 and S4). The spatial distribution of voxels CSM and CTM appeared to be more complex and variable across subjects (Figure 6 for the group and Figure S5 and S6 for all individual subjects). However, the group data and the majority of the individual subjects suggested distinct regional sensitivities to modulation frequencies (see schematic summary in Figure 7). In both hemispheres, clusters with a preference for fine spectral modulations (high CSM, purple colors) were primarily and consistently localized along the HG and anterior superior temporal gyrus (STG) (see circles on group maps - Figure 6), while clusters with a preference for coarse spectral modulations (low CSM, orange color) were mostly located posterior-laterally to HG, on the planum temporal (PT) and on STG (see squares on group maps – Figure 6). Bilaterally, a preference for slow temporal modulations (low CTM, orange color) was found along HG and STG, whereas clusters with a preference for fast temporal modulations (high CTM, purple) were observed on the PT, posteriorly to HG and in a region medially adjacent to HG. Supporting the spatial dissociation between spectral and temporal modulation at map level, we found a significant negative correlation between voxels characteristic spectral and temporal modulation (3T: mean [SE] = −0.19 [0.01], p = 0.02; 7T: mean [SE] = −0.11 [0.01], p = 0.01; group level random effects two-tailed t test, see Materials and Methods).

## Discussion

### Mechanisms of spectral and temporal modulation processing

Our results show that the representation of natural sounds in the human auditory cortex relies on a frequency-specific analysis of combined spectro-temporal modulations. By showing superior performance of the joint MTF-based model over the independent model, we have demonstrated that the hypothesis of independent tuning for spectral [16] and temporal modulations [30] is insufficient to account for the representation of natural sounds in the human auditory cortex. Furthermore, the frequency-specificity that we revealed indicates that the organization of the auditory cortex according to frequency extends beyond the representation of the spectral content of incoming sounds. We show that, at least for spectro-temporal modulations, the integration along the whole range of frequencies occurs at a later stage than the extraction of the feature itself.

The encoding mechanism that our results support is consistent with a recent study showing that a frequency-specific representation of combined spectro-temporal modulations allows the accurate reconstruction of speech in the human posterior superior temporal gyrus [31]. The present study generalizes these observations to sounds from natural categories other than speech. Furthermore, our results are in line with psychophysics studies showing that tuning for combined spectro-temporal modulations provides a better account of human behavior during the performance of auditory tasks [32], [33].

Previous neuroimaging studies had examined the processing of spectral and temporal modulations by measuring the tuning to synthetic stimuli with varying spectral modulation frequency, temporal modulation frequency or the combination of the two. This approach suffers from two main limitations. First, natural sounds are complex stimuli with characteristic statistical regularities [6], [34]–[36] and it has been suggested that the auditory system is adapted to such regularities in order to efficiently encode sounds in natural settings [37]. Even the most complex synthetic stimuli lack both the statistical structure and the behavioral relevance of natural sounds; therefore there is not guarantee that they engage the auditory cortex in processing that is actually used during the analysis of natural sounds. Second, tuning per se only allows indirect inference on cortical encoding mechanisms: proofing a general computational strategy requires building a model that is able to predict brain responses to a broad range of natural stimuli [38]. The approach that we followed in the present study allowed overcoming these limitations, therefore providing direct evidence for a specific encoding mechanism. However, two important caveats should be mentioned. First, by estimating a linear mapping between modulation acoustic space and fMRI responses, we only modeled the linear response properties of voxels. One might argue that because of the linear approximation, the use of natural sounds provides no advantage over synthetic stimuli (e.g. dynamic ripples). However, it has been shown that tuning properties of both auditory [39]–[41] and visual [42], [43] neurons differ significantly under natural and synthetic stimulus condition and that linear models obtained from natural stimuli predict neurons responses significantly better. This shows that natural and synthetic stimuli activate neurons in a different manner and that, despite being an incomplete description, linear models estimated from responses to natural stimuli may be more accurate. We suggest that this is true also for models of voxels receptive fields. Second, it might be possible that some auditory cortical locations are selective to higher-level sound attributes (i.e. sound categories) that co-occur with specific spectro-temporal modulations. As a consequence of this co-occurrence, these locations would then be assigned with a preferred temporal and spectral modulation frequency, only in virtue of their category selectivity. To examine the role of category selectivity on our results, we performed additional analyses on the 7T dataset and tested a model that included categorical predictors together with the original MTF-based model (Text S1). The results showed that predictions of new sounds do not improve with the inclusion of categorical information (mean [SE] = 0.76 [0.03]) and that estimated CTM and CSM maps do not change (Figure S8). This analysis suggests that category tuning may result from preference to specific lower level features or combination of features. However, it would be important to further investigate this issue and compare responses and voxels receptive fields obtained with both natural and synthetic sounds (see [27] for a similar comparison for frequency responses). Such an investigation is experimentally challenging, as it would require as many stimuli (dynamic ripples) as model parameters used in the present study. However, it could be crucial for understanding the relation between acoustic and perceptual levels of sound representation in the auditory cortex.

### Spatial topographies and relation to current functional models

On the basis of positron emission tomography responses to tone sequences that differed either in the temporal or spectral dimension, Zatorre and Belin [44] reported a left-hemispheric preference for rapid temporal processing and complementary preference in the right hemisphere for fine-grained spectral analysis. While the analyses we conducted cannot exclude that hemispheric differences exist at regional level, our maps - obtained at a much higher spatial resolution and with natural sounds - suggest a more complex spatial pattern of spectral and temporal modulation preference within each hemisphere. The most evident characteristic is that – in both the hemispheres - regions located posterior-laterally to HG (see squares in Figure 6 and the schematic summary in Figure 7) preferably encode coarse spectral information with high temporal precision while regions located along HG or antero-ventrally (see circles in Figure 6 and the schematic summary in Figure 7) preferably encode fine-grained spectral information with low temporal precision. Both the two previous human neuroimaging studies that investigated tuning for combined spectro-temporal modulations with dynamic ripples ([20], [21]) reported a role of anterior auditory regions in the analysis of fine spectral details, which is consistent with our observations, whereas results are less coherent for temporal modulation maps. Again, a direct comparison between maps obtained with dynamic ripples and natural sounds would be required to address this issue.

Our results of spatial topographies for CTM and CTF support the view that the auditory cortex forms multiple (parallel) representations of the incoming sounds at different spectro-temporal resolutions ([45], [46]). We suggest that this may be relevant for enabling flexible behavior, as different goal-oriented sound processing may benefit from different types of auditory representations. Importantly, this suggestion can be tested empirically in future experiments and studies where (natural) sounds are presented in the context of multiple behavioral tasks.

A spectral-temporal resolution “trade-off” analogous to the one reported here has previously been described for neurons in the inferior colliculus of the cat [47], [48] and is in agreement with the low-pass behavior of the MTF of the human auditory cortex [21] and the psychophysically derived detection thresholds for spectro-temporal modulations [9]. Furthermore, modulation spectra of natural sounds exhibit a similar trade-off, i.e. natural sounds rarely present both high spectral and high temporal modulation frequencies [6], [10]. A match between stimulus statistics and neuronal response properties is generally interpreted as an evidence for the theory of efficient coding [19], [36], [37], [48], [49]. Thus, our data provide further support to the idea that the auditory system has adapted in order to efficiently encode the statistical regularities of natural sounds.

### Comparing computational models of auditory processing with fMRI

Besides providing insights into the representation of natural sounds in the human auditory cortex, our results pave the way to future research aiming at testing increasingly complex encoding models of auditory processing. The combination of fMRI and “encoding” techniques has proven to be a successful tool to investigate the representation of natural images in the human visual cortex [24], [50], [51], as well as to predict the brain activity associated with the meaning of words [52]. In the auditory domain, the application of such powerful method has lagged behind. We have recently demonstrated that “encoding” makes it possible to detect the spectral tuning of voxels in the human auditory cortex from fMRI responses to natural sounds [27]–[29]. In the present study, we show that models embedding more complex representations than frequency selectivity can be learned from fMRI activity. The challenge for future studies is to explore more sophisticated voxels receptive field models. Here we only considered voxels tuning along three stimulus dimensions (frequency, spectral modulations and temporal modulations). However, natural sounds vary in a higher dimensional acoustic space and interactions with parameters not considered here might occur.

Interestingly, we consistently observed higher prediction accuracy for the 7T compared to the 3T dataset (Figure 4), despite the fact that at 7T the model was trained and tested on independent sound ensembles (while different presentations of the same sounds were used for the 3T data set). We interpret this difference as a result of the interplay between two important factors, namely the number of stimuli and the functional contrast to noise ratio (CNR). The larger amount of different sounds employed in the 7T experiment has probably increased the variance along the dimensions represented by the model; this, together with the higher CNR and the higher spatial specificity achieved at 7T, has likely led to a more accurate model estimation, which in turn has resulted in higher prediction accuracy. These observations provide important guidelines for the design of future experiments in this framework.

It should be mentioned that in our study, accuracy based on percent correct was significantly above chance ([12.5%, 12.5%, 16.7%, 20.8%, 25%] for subjects S6–S10 for the best performing model at 7T; chance = 4.2%), but still quite small compared to the outstanding results reported in similar encoding studies in the visual domain (e.g. [24]). However, the distribution of ranks was skewed towards 1 (correct identification), indicating that for most sounds the correlation between predicted and measured response was ranked very high (e.g. second or third). The lower percent correct performance for sound identification can be ascribed to a variety of reasons. It might be due to the lower functional CNR, as BOLD responses observed in the auditory cortex are substantially lower than those in the visual cortex, probably because of the effects of the scanner noise [53]. Furthermore, our clustered fMRI acquisition with a silent gap between scans limits the number of sounds used for training/testing the model (compared e.g. to the number of images in [24]). Finally, the model of receptive field based on spectro-temporal modulations might be too simple for allowing distinguishing two acoustically similar sounds (e.g. two speech sounds).

Although the proposed combination of high field fMRI with the encoding approach is valuable for testing well-defined hypotheses on sound processing in the human brain, there are intrinsic limitations. A voxel - even at the high spatial resolution achievable with 7T fMRI - samples a large number of neurons and the relation between the measured BOLD signal and the neural activation is only partly understood. Results based on BOLD fMRI (and thus fMRI encoding) reflect a complex mixture of neuronal (spiking and synaptic activity, excitation, inhibition) as well as neurovascular phenomena. In particular, neural inhibition may be associated with both positive and negative BOLD, depending on the specific neural network configuration [54]. Understanding the neuronal dynamics underlying our fMRI observations would thus require combining electrophysiological (at single-cell and neuronal population level) and fMRI investigations in animal models [55] and/or humans [40].

In summary, our study represents a first demonstration of how fMRI data and “encoding” techniques can be successfully combined to test competing computational models of auditory processing and to concurrently estimate response properties of cortical locations along multiple dimensions within an ecologically valid framework. Also, by using a biologically inspired computational model, we pave the way for linking electrophysiology in animals and non-invasive research in humans.

## Materials and Methods

### Ethics statement

The Ethical Committee of the Faculty of Psychology and Neuroscience at Maastricht University and the Institutional Review Board for human subject research at the University of Minnesota granted approval for the study at 3T and 7T respectively.

### Experimental procedure

Subjects, stimuli, experimental design, MRI parameters, and data preprocessing have been reported in previous publications from our group [27]–[29] (see Text S1). In the following, the most relevant details of the experimental design will be briefly described.

We used 60 (168) recordings of natural sounds for the 3T (7T) experiment. Stimuli included human vocal sounds (both speech and non-speech, e.g., baby cry, laughter, coughing), animal cries (e.g., dog, cat, horse), musical instruments (e.g., piano, flute, drums), scenes from nature (e.g., rain, wind, thunder), and tool sounds (e.g., keys, scissors, vacuum cleaner). Sounds were sampled at 16 kHz and their duration was cut at 1000 ms. Sound onset and offset were ramped with a 10 ms linear slope, and their energy (RMS) levels were equalized.

The 3T and 7T experiments consisted of 3 and 8 runs, respectively; in the 3T (7T) experiment, each run lasted approximately 25 (10) minutes. In the 7T experiment, data were subdivided into six train runs and two test runs. In the train runs, 144 of the 168 stimuli were presented with 3 repetitions overall (i.e. each sound was presented in 3 of the 6 train runs). The remaining 24 sounds were presented in the test runs and repeated 3 times per run.

Sounds were presented in the silent gap between acquisitions with a randomly assigned inter-stimulus interval of 2, 3, or 4 TRs - plus an additional random jitter. Zero trials (trials where no sound was presented; 10% of the trials in the 3T experiment; 6% (5%) of the trials in train (test) runs in the 7T experiment), and catch trials (trials in which the sound which was just heard was presented; 6% of the trials in the 3T experiment; 6% (3%) of the trials in train (test) runs in the 7T experiment) were included. Subjects responded with a button press when a sound was repeated. Catch trials were excluded from the analysis.

### Joint frequency-specific MTF-based model

The stimulus representation in the modulation space was obtained as the output of a biologically inspired model of auditory processing [22], that explicitly encodes the modulation content of a sound spectrogram. The auditory model consists of two main components: an *early* stage that accounts for the transformations that acoustic signals undergo in the early auditory system, from the cochlea to the midbrain; and a *cortical* stage that simulates the processing of the acoustic input at the level of the (primary) auditory cortex. The spectral analysis performed by the cochlea is mimicked by a bank of 128 overlapping bandpass filters with constant-Q (Q_10 *dB*_ = 3), equally spaced along a logarithmic frequency axis over a range of 5.3 oct (*f* = 180–7040 Hz). The output of each filter enters a hair cell stage, where it undergoes high-pass filtering, optional non-linear compression and low-pass filtering. A midbrain stage models the enhancement of frequency selectivity as a first-order derivative with respect to the frequency axis, followed by a half-wave rectification. Finally, a short-term temporal integration (time constant *τ* = 8 ms) accounts for the loss of phase locking observed in the midbrain. The auditory spectrogram generated by the early stage is further analyzed by the cortical stage, where neurons are modeled as 2-dimensional (2D) modulation selective filters that are tuned to a specific combination of spectral and temporal modulations, and operate over a limited range of frequencies along the tonotopic axis. These filters have constant Q and are directional, i.e. they respond either to upward or downward frequency sweeps. Computationally, the cortical filter bank performs a complex wavelet decomposition of the auditory spectrogram. The magnitude of such decomposition yields a phase-invariant measure of modulation content. Ultimately, the model's output is a multi-resolution representation of the spectrogram envelope as a function of time, frequency, spectral and temporal modulations, and directionality.

We derived the auditory spectrogram and its modulation content using the “NSL Tools” package (available at http://www.isr.umd.edu/Labs/NSL/Software.htm) and customized Matlab code (The MathWorks Inc.). Pilot analyses showed that model performance was not significantly affected by changes in the parameters of the early stage. Accordingly, parameters for the spectrogram estimation were fixed (i.e. not estimated in the fitting procedure) and set as described above and in [22]. The modulation content of the auditory spectrogram was computed through a bank of 2D modulation selective filters tuned to spectral modulation frequencies of *Ω* = [0.5, 1, 2, 4] cyc/oct and temporal modulation frequencies of *ω* = [1, 3, 9, 27] Hz. The filter bank output was computed at each frequency along the tonotopic axis and then averaged over time. In order to avoid overfitting, a reduced modulation representation was obtained as follows (3T: 3 tonotopic frequencies×4 spectral modulations×4 temporal modulations = 48 parameters to learn; 7T: 8 tonotopic frequencies×4 spectral modulations×4 temporal modulations = 128 parameters to learn; note that we chose a different number of parameters for the 3T and 7T datasets due to the different number of stimuli used for model's estimation - 60 and 144 stimuli, respectively). First, the time-averaged output of the filter bank was averaged across the upward and downward filter directions (note that this corresponds to assuming that sweep direction does not affect voxels activation levels). Then, we divided the tonotopic axis in ranges with constant bandwidth in octaves and averaged the modulation energy within each of these regions. We defined three frequency ranges in the 3T experiment and eight in the 7T experiment. The above processing steps were applied to all stimuli, resulting into an [*S×N*] feature matrix **F** of average modulation energy, where *S* is the number of sounds, and *N* is the number of features in the reduced modulation representation.

### Tonotopy model

The stimuli representation in the frequency space was obtained using only the input stage of the auditory model. The spectrogram was computed at 128 logarithmically spaced frequency values (*f* = 180–7040 Hz) and averaged over time. In the 3T experiment, we generated a reduced frequency representation in order to restrain the effects of overfitting (note that in the 7T experiment the number of observations in the train set was already higher than the number of parameters to estimate). We divided the tonotopic axis in 48 bins with constant bandwidth in octaves and averaged the frequency content within each of these regions. We chose 48 bins in order to have the same number of parameters for both the MTF-based and the tonotopy model. The above processing steps were applied to all stimuli, resulting into an [*S×N*] feature matrix **F** of time-averaged frequency content, where *S* is the number of sounds, and *N* is the number of frequency bins.

### Joint frequency non-specific MTF-based model

We generated the non-localized modulation representation by averaging the frequency-specific joint representation along both time and frequency (this is similar to performing a 2D Fourier transform of the spectrogram). This resulted in a representation with 16 features (4 temporal modulations×4 spectral modulations). However, frequency specific information is indeed reflected in voxels' activity [26], [27]; therefore, we concatenated the modulation representation with a tonotopic representation obtained as described above for the tonotopy model. We employed 32 frequency bins for the 3T dataset and 112 for the 7T dataset, resulting in a final representation with 48 and 128 features, respectively.

### Independent frequency-specific MTF-based model

We generated the independent modulation representation by filtering the auditory spectrogram with one-dimensional purely spectral and purely temporal modulation filters. Filters were tuned to spectral modulation frequencies of *Ω* = [0.5, 1, 2, 4] cyc/oct and temporal modulation frequencies of *ω* = [1, 3, 9, 27] Hz. The output of each filter bank was averaged over time and within frequency ranges with constant bandwidth in octaves. In order to have a representation with the same number of features as for the joint model, we defined 6 frequency ranges in the 3T experiment and 16 in the 7T experiment. Finally, the outputs of the purely spectral and purely temporal filter banks were concatenated, resulting in a representation with 48 features for the 3T dataset (6 tonotopic frequencies×4 temporal modulations+6 tonotopic frequencies×4 spectral modulations) and 128 for the 7T dataset (16 tonotopic frequencies×4 temporal modulations+16 tonotopic frequencies×4 spectral modulations). The above processing steps were applied to all stimuli, producing an [*S×N*] feature matrix **F** of average modulation energy, where *S* is the number of sounds, and *N* is the number of features.

### Independent frequency non-specific MTF-based model

We generated the non-localized independent representation by averaging across frequency the frequency-specific independent representation. This resulted in a representation with 8 features (4 temporal modulations+4 spectral modulations). The final model was obtained by concatenating the modulation representation with a tonotopic representation obtained as described above for the tonotopy model. We employed 40 frequency bins for the 3T dataset and 120 for the 7T dataset, resulting in a final representation with 48 and 128 features, respectively.

### Model estimation and evaluation

In the 7T experiment, independent train and test runs involving two completely distinct sound sets were used to train and assess the model, whereas leave run out cross-validation was performed for the 3T dataset (the final model parameters and the overall prediction accuracy were computed as the average across cross validations).

### Estimation of fMRI responses to natural sounds

For each voxel *i*, the response vector *Y_i_* [(*S*×*1*), *S* = number of sounds] was obtained in two steps. First, a deconvolution analysis with all stimuli treated as a single condition was used to estimate the hemodynamic response function (HRF) common to all stimuli. Then, using this HRF and one predictor per sound, we computed the beta weight of each sound [56]. Further analyses were performed on voxels with a significant response to the sounds (p<.05, uncorrected in order not to be too stringent at this stage of the process) within an anatomically defined mask, which included HG, HS, PT, PP, and STG.

### Estimation of model parameters

The fMRI activity *Y_i_* [*S_train_×1*] at voxel *i* was modeled as a linear transformation of the feature matrix **F***_train_* [*S_train_×N*] plus a noise term *n* [*S_train_×1*] as follows:
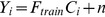
(1)where *S_train_* is the number of sounds in the training set, and *C_i_* is an [*N×1*] vector of model parameters, whose elements *c_ij_* quantify the contribution of feature *j* to the overall response of voxel *i*. Note that Equation 1 does not include a constant term as columns of matrices **F***_train_* and Y_i_ were converted to standardized z-scores. Z-scoring of the features and responses does not affect the expressive capacity of the linear regression model. However, in a regularized regression framework like ridge regression (see below), z-scoring does affect the estimated model parameters (weights). In the present study, z-score was performed because the energy content of natural sounds varies on different scales across frequencies and modulations. As a consequence, the estimated model parameters would not be comparable without performing the z-score normalization.

The solution to Equation 1 was computed using *ridge regression* [57]. The regularization parameter λ was determined independently for each voxel by automatically inspecting the stability of the ridge trace, that is changes in the parameter estimates as a function of λ [58]. Namely, parameter estimates 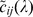 were obtained for a range of increasing λ values [λ_1_, λ_2_, …, λ_p_], and the regularization parameter was set at the value λ^*^ where all parameter estimates consistently changed less than 20% of their initial value 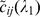:
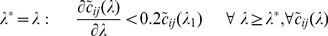
(2)The inspection of the ridge trace represented an advantage in terms of trade-off between accurate model estimation and computational load. Namely, we observed that the selection of the regularization parameter via cross validation was computationally slower, while not yielding any significant improvement on models performance.

### Model evaluation

We quantified model's prediction accuracy by performing a sound identification analysis [24]. Namely, we used the fMRI activity patterns predicted by the estimated models to identify which sound had been heard among all sounds in the test set.

Because model parameters were estimated in z-score units, we converted to standardized z-score the columns of the feature and response matrices for the stimuli in the test set. Given the trained model 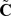 [*N×V*] (where V is the number of voxels), and the feature matrix **F***_test_* [*S_test_×N*] for the test set, the predicted fMRI activity 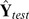 [*S_test_×V*] for the test sounds was obtained as follows:
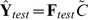
(3)Then, for each stimulus *s_i_* we computed the correlation between its predicted fMRI activity 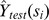 [*1×V*] and all measured fMRI responses 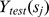 [*1×V*], j = 1, 2, …, S. The rank of the correlation between predicted and observed activity for stimulus *s_i_* was selected as a measure of the model's ability to correctly match 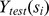 with its prediction 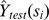. The matching score *m* for stimulus *s_i_* was obtained by normalizing the computed rank between 0 and 1 as follows (*m* = 1 indicates correct match; *m* = 0 indicates predicted activity pattern for stimulus *s_i_* was least similar to the measured one among all stimuli):
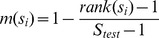
(4)Normalized ranks were computed for all stimuli in the test set, and the overall model's accuracy was obtained as the mean of the matching scores across stimuli. Note that the metric we used (Eq. 4) generalizes the more straightforward percent correct, a rank-based metric that considers only stimuli that are ranked first, i.e. stimuli that are correctly identified. Percent correct is a comprehensive metric when models identify new stimuli with high accuracies (close to 100%). As this was not the case in our data (see Discussion), it is informative to look at the whole distribution to assess the degree of incorrect identification.

Statistical significance of the observed accuracy was assessed with permutation testing. Specifically, the empirical null-distribution of accuracies was obtained by randomly permuting (*P* = 200 permutations) the stimulus labels (i.e. *S* in matrix **Y**) and repeating the training and testing procedures. In order to preserve the spatial correlations among cortical locations, the same permutations were applied to all voxels. The regularization parameter was constant across permutations and was set to the value derived when the model was estimated on the unpermuted set of responses. When compared by means of t-test, accuracies were converted to z-scores via Fisher's transformation in order to reduce deviations from normality.

### Topographic maps of temporal modulation, spectral modulation, and frequency preference

For all voxels, response profiles for temporal modulation, spectral modulation and frequency were computed as marginal sums of the estimated stimulus-activity mapping function **C** of the joint frequency-specific MTF-based model, as follows:
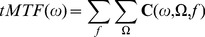
(5)
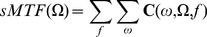
(6)
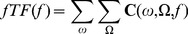
(7)where *tMTF* and *sMTF* are the temporal and spectral modulation transfer functions, respectively, and *fTF* is the frequency transfer function. Voxels characteristic values (CTM, CSM, CF) were defined as the point of maximum of the *tMTF*, *sMTF* and *fTF*, respectively. A continuous representation of preferred values was obtained by spatial smoothing using a 2-neighbor (3-neighbor) voxels filter for the 3T (7T) dataset. Cortical maps were generated by color-coding the voxels' preferred values and projecting them onto an inflated representation of the subject's cortex. Individual maps were subsequently transformed to functional cortex based aligned (fCBA) space (see below) where group maps were obtained as the mean across subjects. Only voxels that had been included in the analysis of at least 3 out of the 5 subjects were considered when computing group maps.

To assess the reliability of the estimated voxels tuning preference, we computed the signal-to-noise ratio (SNR) of the MTFs estimates via a bootstrap resampling procedure applied to all individual subjects (see Text S1 and Figure S7).

### Relation between voxels characteristic spectral and temporal modulation

For each subject, we computed the Spearman's rank correlation coefficient between voxels characteristics CSM and CTM (prior to spatial smoothing). In order to take into account any possible bias introduced by the model's estimation procedure, we derived the empirical expected value of no correlation by computing the correlation coefficient between voxels CSM and CTM as obtained after permuting the stimulus labels (see above). Statistical significance of the Fisher-transformed correlation coefficients was assessed via a group level random effect two-tailed t test.

### Functional cortex based alignment

Additionally to the main experiments, localizer data were collected as responses to amplitude modulated tones (see Text S1). Tonotopy maps were computed with best-frequency mapping [26], and resulting maps were used for fCBA [59] as follows. In each subject and hemisphere, we delineated the low frequency region consistently present in the vicinity of Heschl's gyrus as region of interest. FCBA was partially driven by this functional region (weighting decreased over iterations), and partially by anatomical information (weighting increased over iterations; [60]). The resulting alignment information was used for calculating and displaying group cortical maps.

## Supporting Information

Figure S1 *Joint* and *independent* modulation representations. Spectrograms illustrate a schematic of channels in a modulation filter bank. Vertical and horizontal spacing between bars indicate channels preferred spectral (Ω) and temporal modulation frequencies (ω), respectively. (A) In the *joint* representation, the conjunction of spectral and temporal modulations is analyzed by spectro-temporal channels tuned to specific combinations of spectral and temporal modulation frequencies. Direction of bar tilt indicates tuning for upward or downward modulations. (B) In the *independent* representation, spectral and temporal modulations are independently encoded by separate spectral (top) and temporal (bottom) channels.
(TIF)

Figure S2 Schematic of model estimation and evaluation. (A) FMRI responses to a wide variety of natural sounds are used to estimate an encoding model for each voxel. The model projects the stimuli into an N-dimensional feature space and voxels are described as linear combinations of these features. By applying regularized regression, a vector of model's weights is estimated for each voxel. The feature yielding the highest weight is assigned as voxel's characteristic value. (B) Model performance is evaluated by assessing its ability to accurately predict fMRI responses to natural sounds in a new dataset. (S = number of sounds; N = number of features).
(TIF)

Figure S3 Group tonotopic maps. Group maps for the 3T (A) and 7T (B) datasets are displayed on an inflated representation of the group cortex. Maps are shown in the cortical region highlighted by the black square. Group maps are computed as the mean across participants for those voxels that are included in at least 3 individual maps. The black line indicates HG.
(TIF)

Figure S4 Individual tonotopic maps. Individual maps of tonotopy are shown for the 3T (A) and 7T (B) datasets. The black line indicates HG.
(TIF)

Figure S5 Individual topographic maps. Maps of CSM (left) and CTM (right) for all participants in the 3T experiments. Left: purple and orange denote tuning for fine and coarse spectral structures respectively. Right: purple and orange denote tuning for fast and slow temporal variations respectively. The black line indicates HG.
(TIF)

Figure S6 Individual topographic maps. Maps of CSM (left) and CTM (right) for all participants in the 7T experiments. Left: purple and orange denote tuning for fine and coarse spectral structures respectively. Right: purple and orange denote tuning for fast and slow temporal variations respectively. The black line indicates HG.
(TIF)

Figure S7 Stability of MTFs estimates across bootstraps. Single subjects maps of SNR of voxels MTFs as estimated by the joint frequency-specific MTF-based model at 3T (A) and 7T (B). High values of SNR (bright colors) indicate that the estimated MTF is consistent across bootstraps. The black line outlines HG.
(TIF)

Figure S8 Unbiased topographic maps for the 7T dataset. Group maps of CSM, CTM and CF as derived from the joint frequency-specific MTF-based model while explicitly accounting for the effect of sound categories. The black line indicates HG.
(TIF)

Text S1 Supplementary methods and references.
(DOCX)
