# Supplementary figures and images for: Encoding of Natural Sounds at Multiple Spectral and Temporal Resolutions in the Human Auditory Cortex
Source: PLoS Comput Biol. 2014 Jan 2;10(1):e1003412. doi: 10.1371/journal.pcbi.1003412 (PMC3879146; doi:10.1371/journal.pcbi.1003412)

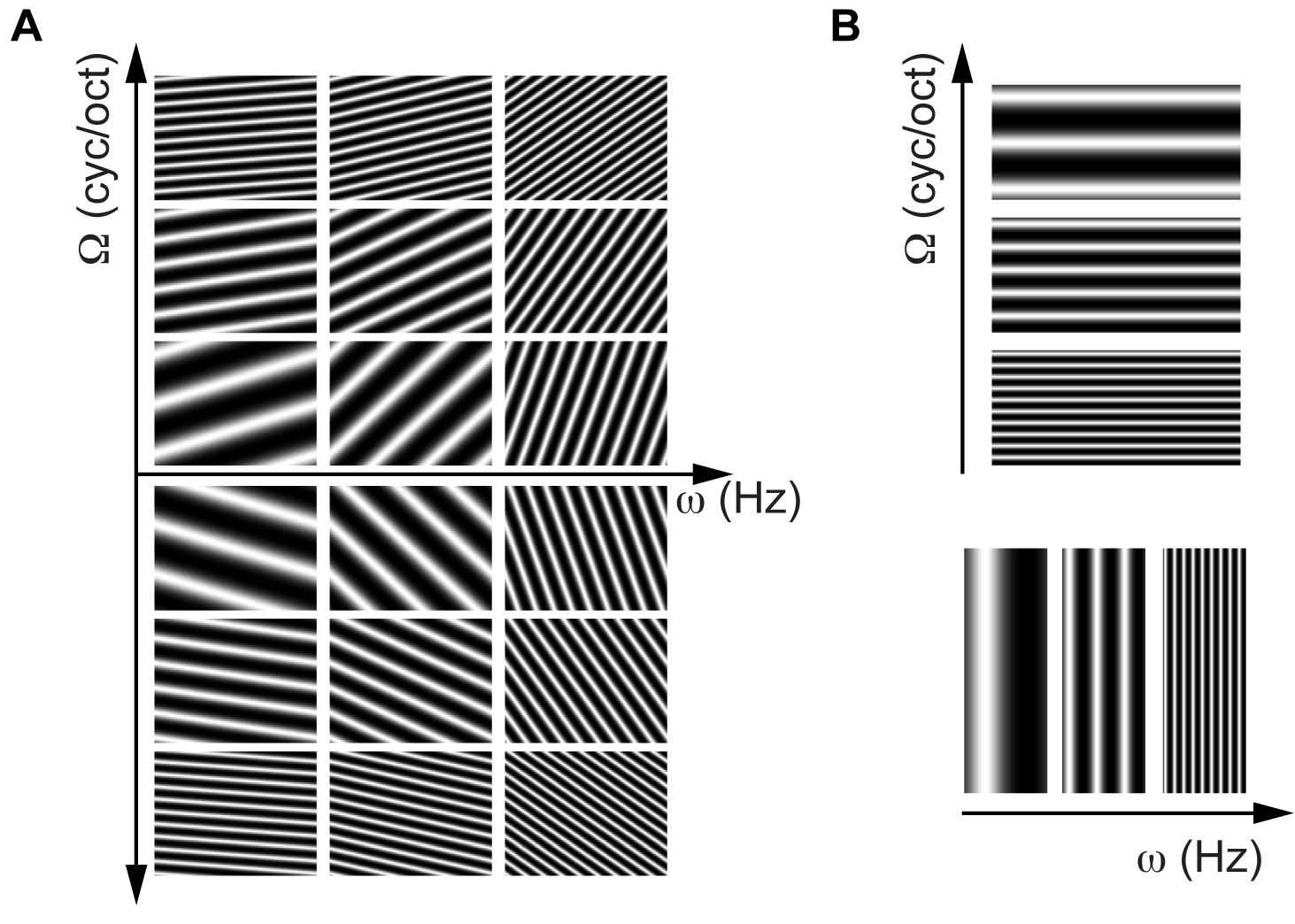

Supplement: Figure S1 — Joint and independent modulation representations. Spectrograms illustrate a schematic of channels in a modulation filter bank. Vertical and horizontal spacing between bars indicate channels preferred spectral (Ω) and temporal modulation frequencies (ω), respectively. (A) In the joint representation, the conjunction of spectral and temporal modulations is analyzed by spectro-temporal channels tuned to specific combinations of spectral and temporal modulation frequencies. Direction of bar tilt indicates tuning for upward or downward modulations. (B) In the independent representation, spectral and temporal modulations are independently encoded by separate spectral (top) and temporal (bottom) channels. (TIF) [file pcbi.1003412.s001.tif]

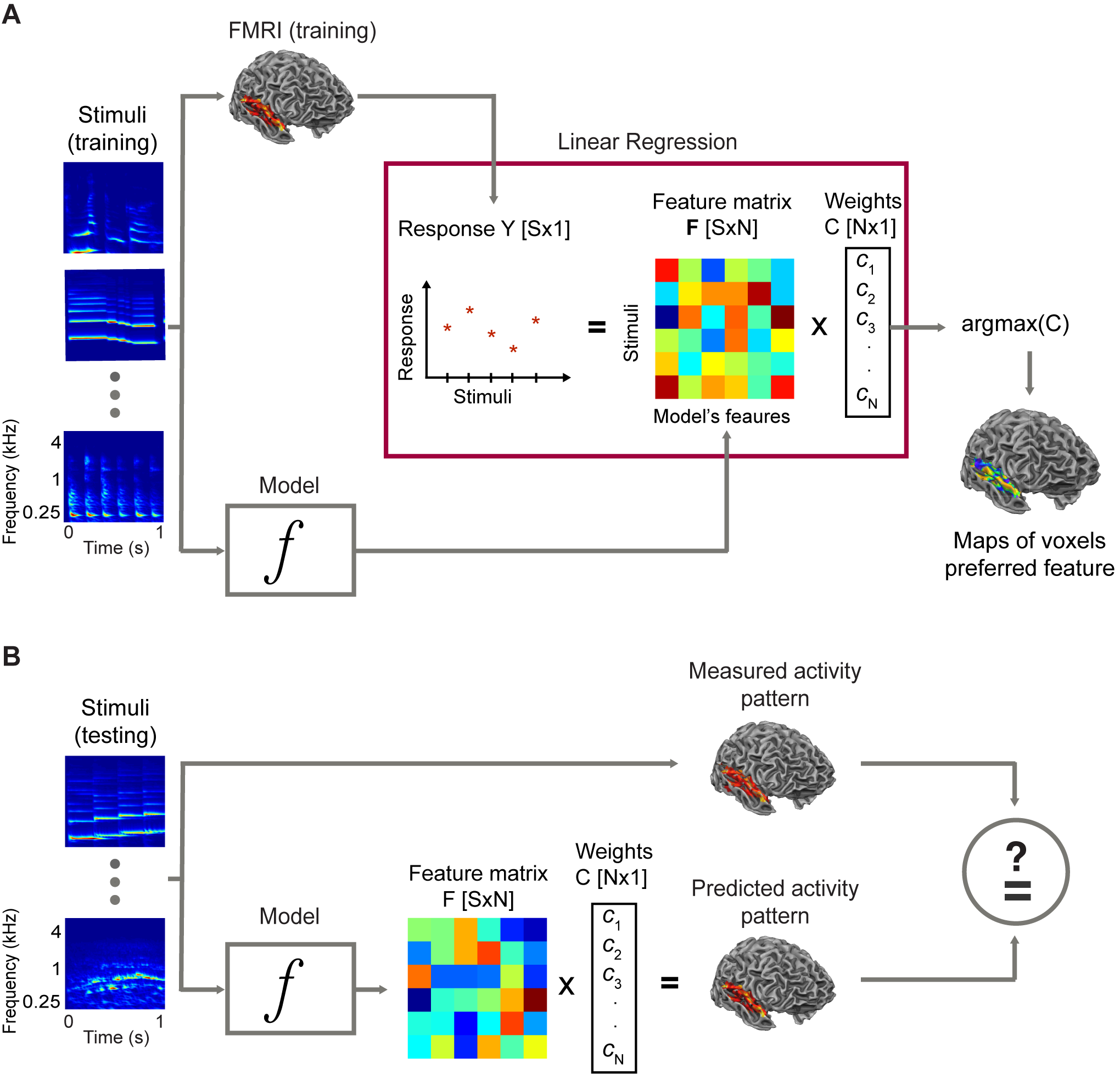

Supplement: Figure S2 — Schematic of model estimation and evaluation. (A) FMRI responses to a wide variety of natural sounds are used to estimate an encoding model for each voxel. The model projects the stimuli into an N-dimensional feature space and voxels are described as linear combinations of these features. By applying regularized regression, a vector of model's weights is estimated for each voxel. The feature yielding the highest weight is assigned as voxel's characteristic value. (B) Model performance is evaluated by assessing its ability to accurately predict fMRI responses to natural sounds in a new dataset. (S = number of sounds; N = number of features). (TIF) [file pcbi.1003412.s002.tif]

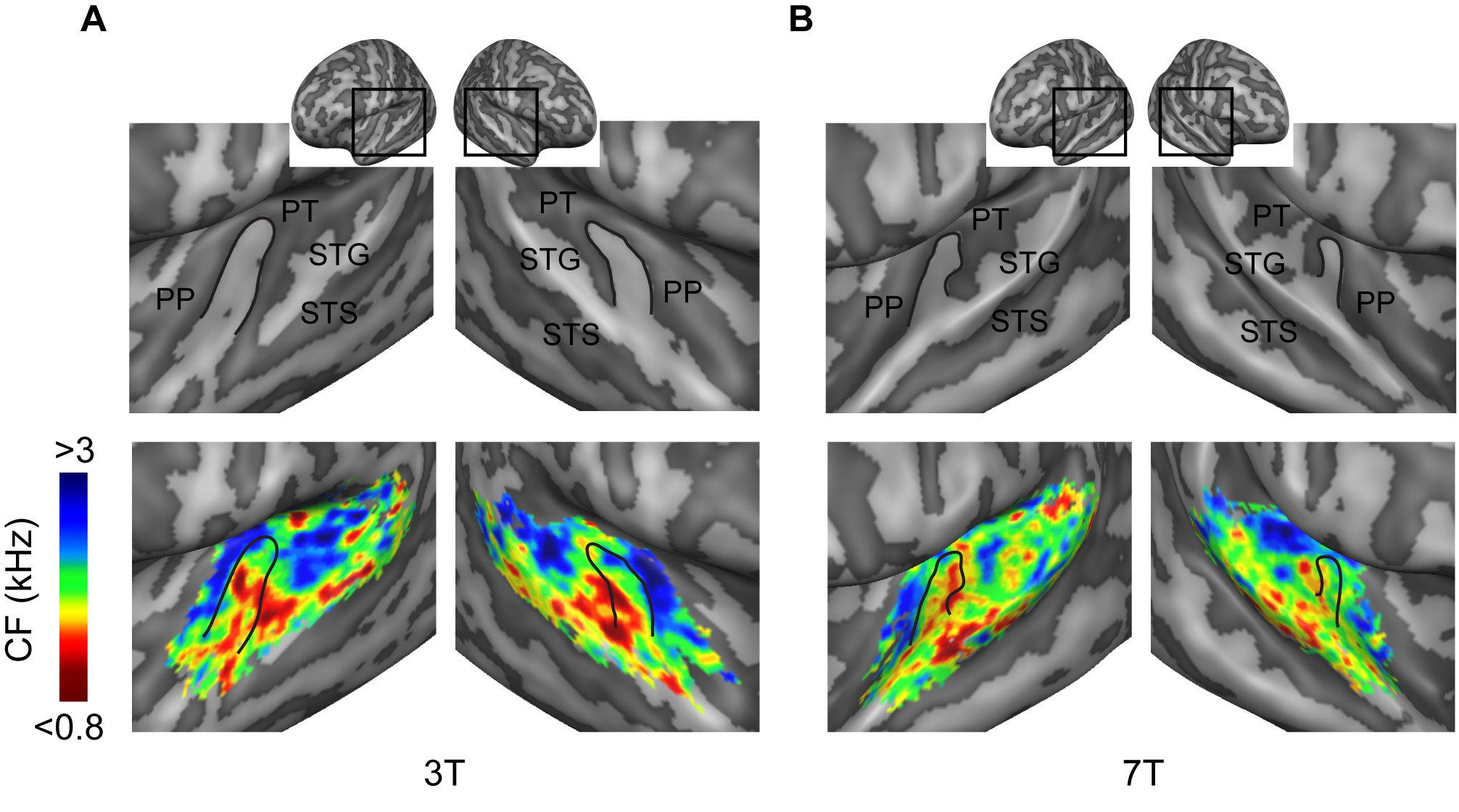

Supplement: Figure S3 — Group tonotopic maps. Group maps for the 3T (A) and 7T (B) datasets are displayed on an inflated representation of the group cortex. Maps are shown in the cortical region highlighted by the black square. Group maps are computed as the mean across participants for those voxels that are included in at least 3 individual maps. The black line indicates HG. (TIF) [file pcbi.1003412.s003.tif]

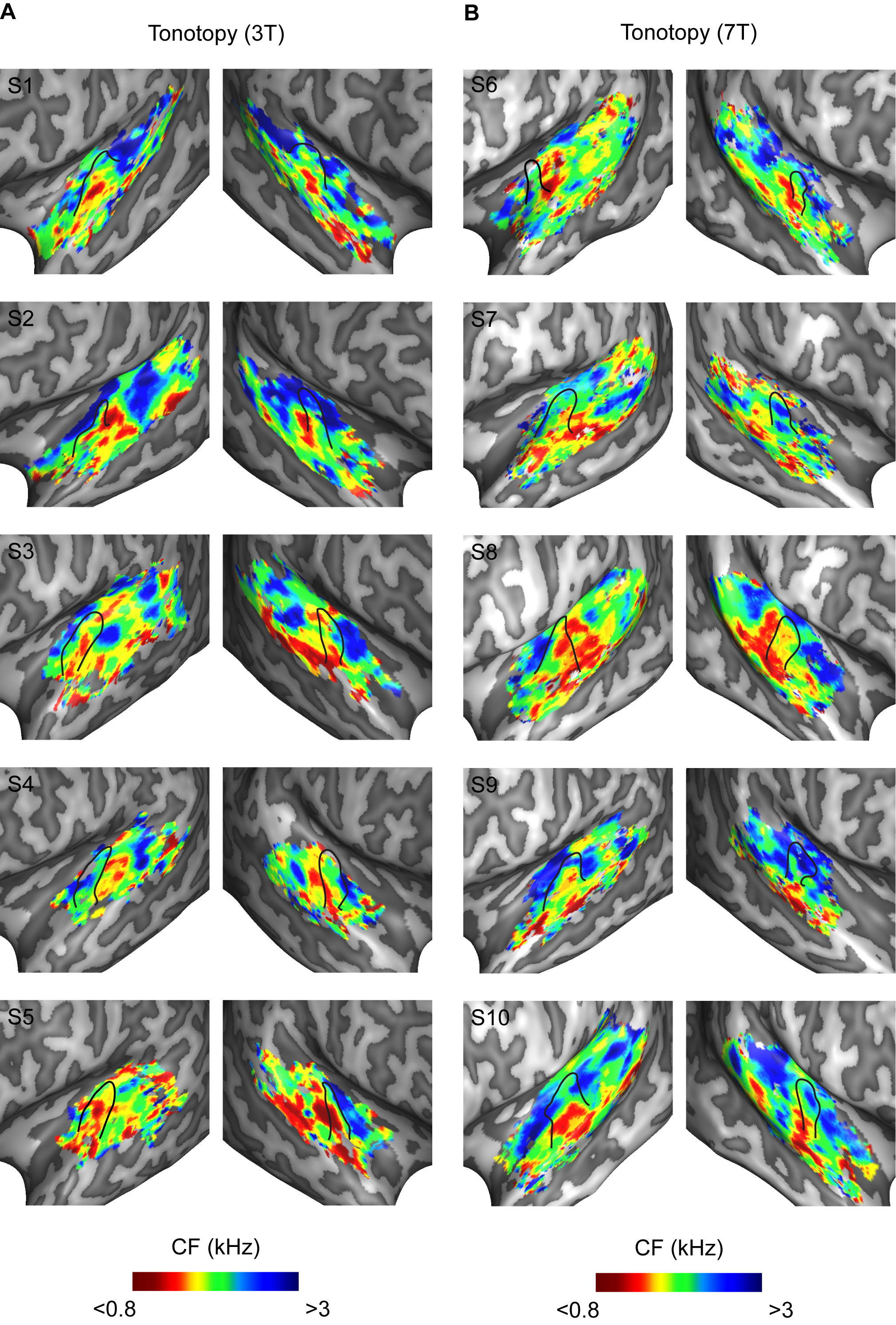

Supplement: Figure S4 — Individual tonotopic maps. Individual maps of tonotopy are shown for the 3T (A) and 7T (B) datasets. The black line indicates HG. (TIF) [file pcbi.1003412.s004.tif]

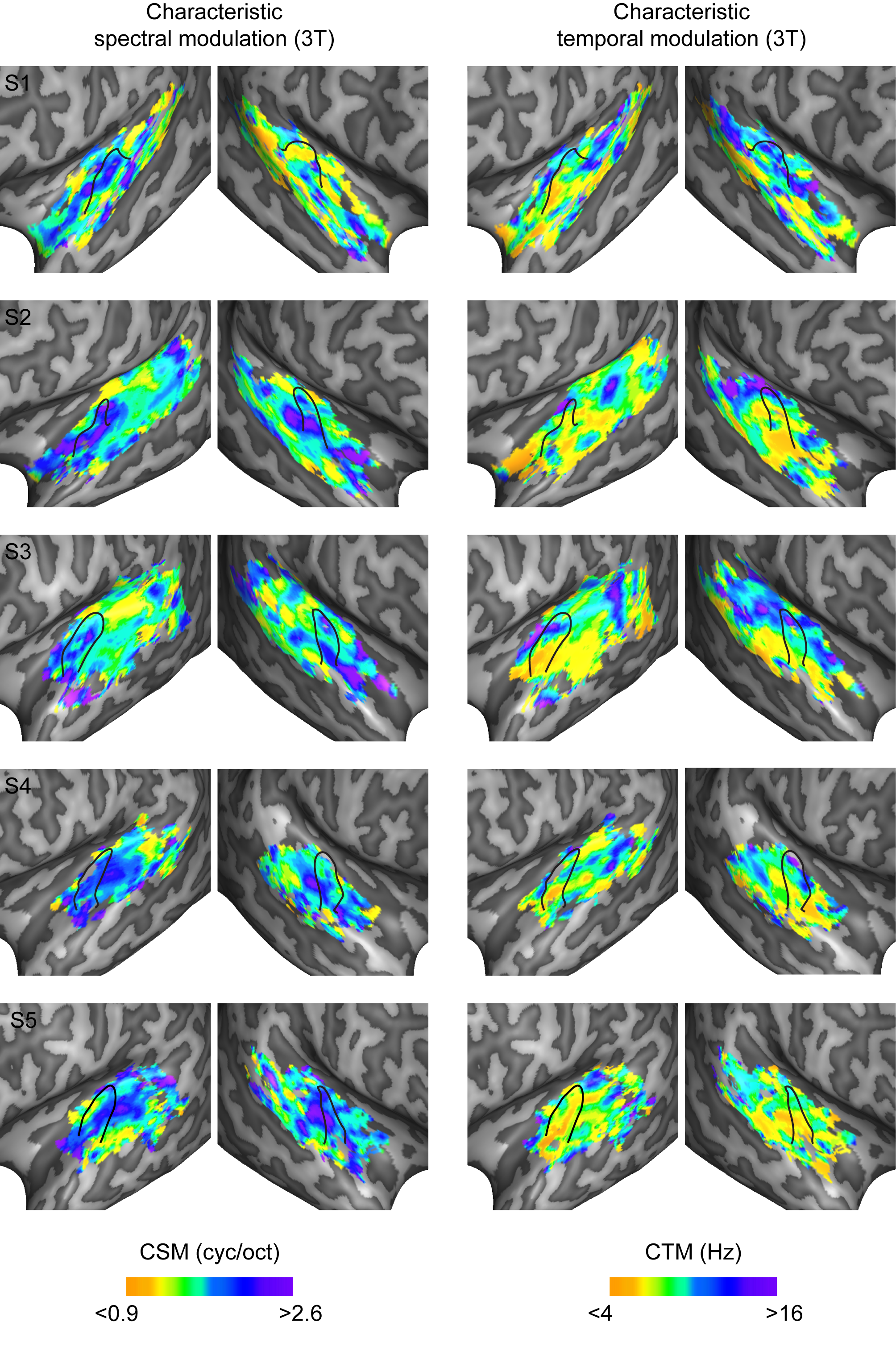

Supplement: Figure S5 — Individual topographic maps. Maps of CSM (left) and CTM (right) for all participants in the 3T experiments. Left: purple and orange denote tuning for fine and coarse spectral structures respectively. Right: purple and orange denote tuning for fast and slow temporal variations respectively. The black line indicates HG. (TIF) [file pcbi.1003412.s005.tif]

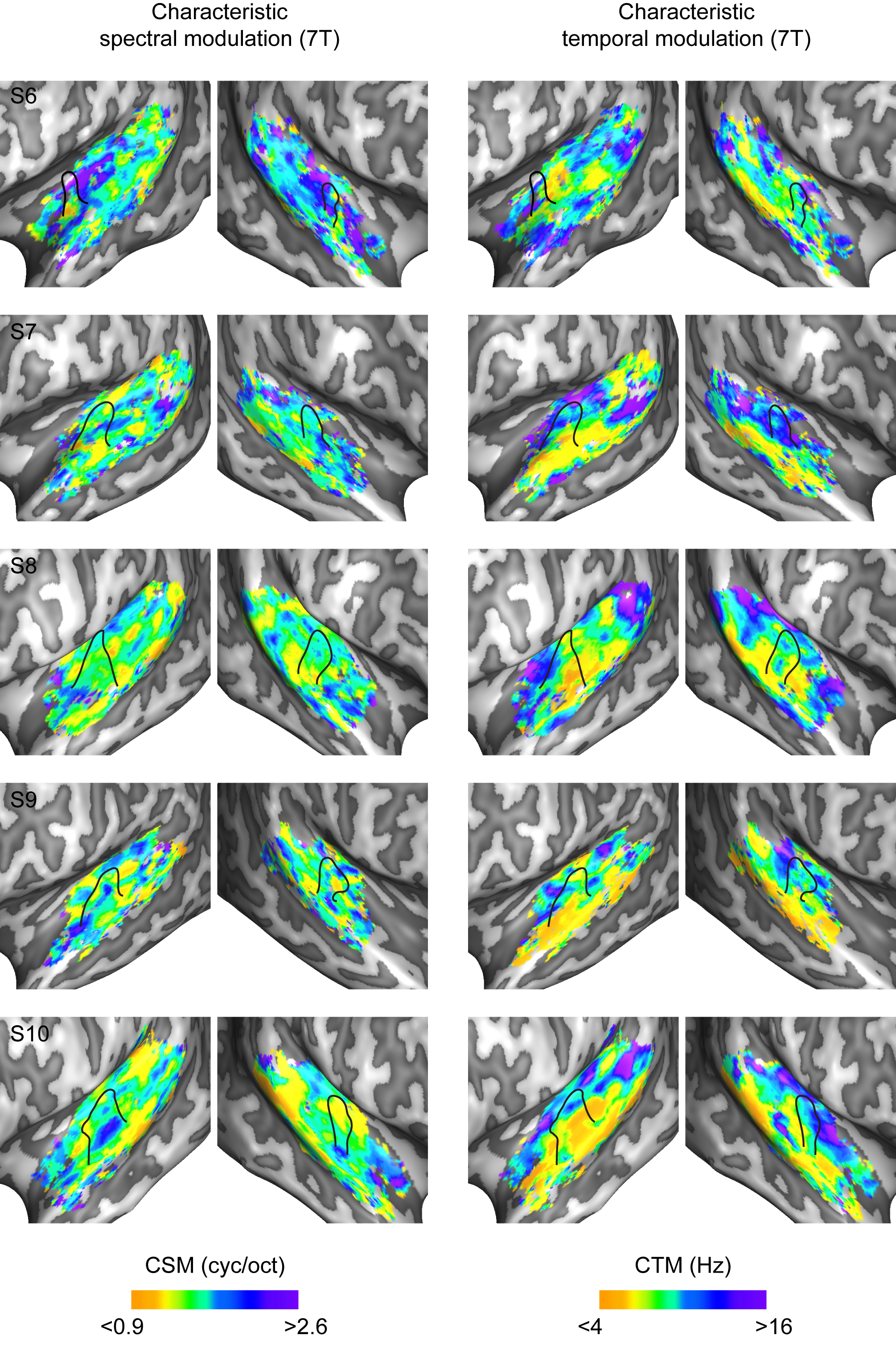

Supplement: Figure S6 — Individual topographic maps. Maps of CSM (left) and CTM (right) for all participants in the 7T experiments. Left: purple and orange denote tuning for fine and coarse spectral structures respectively. Right: purple and orange denote tuning for fast and slow temporal variations respectively. The black line indicates HG. (TIF) [file pcbi.1003412.s006.tif]

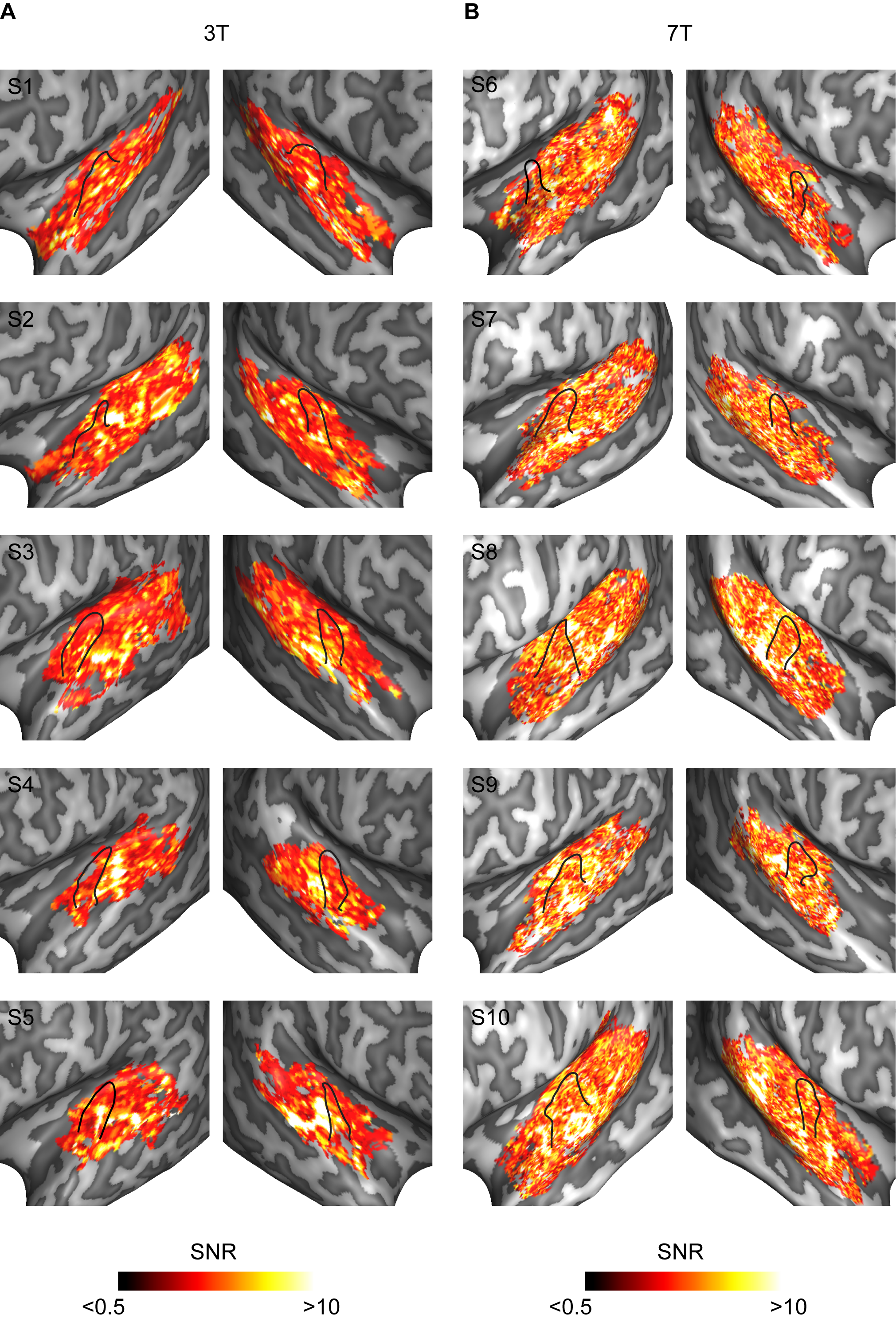

Supplement: Figure S7 — Stability of MTFs estimates across bootstraps. Single subjects maps of SNR of voxels MTFs as estimated by the joint frequency-specific MTF-based model at 3T (A) and 7T (B). High values of SNR (bright colors) indicate that the estimated MTF is consistent across bootstraps. The black line outlines HG. (TIF) [file pcbi.1003412.s007.tif]

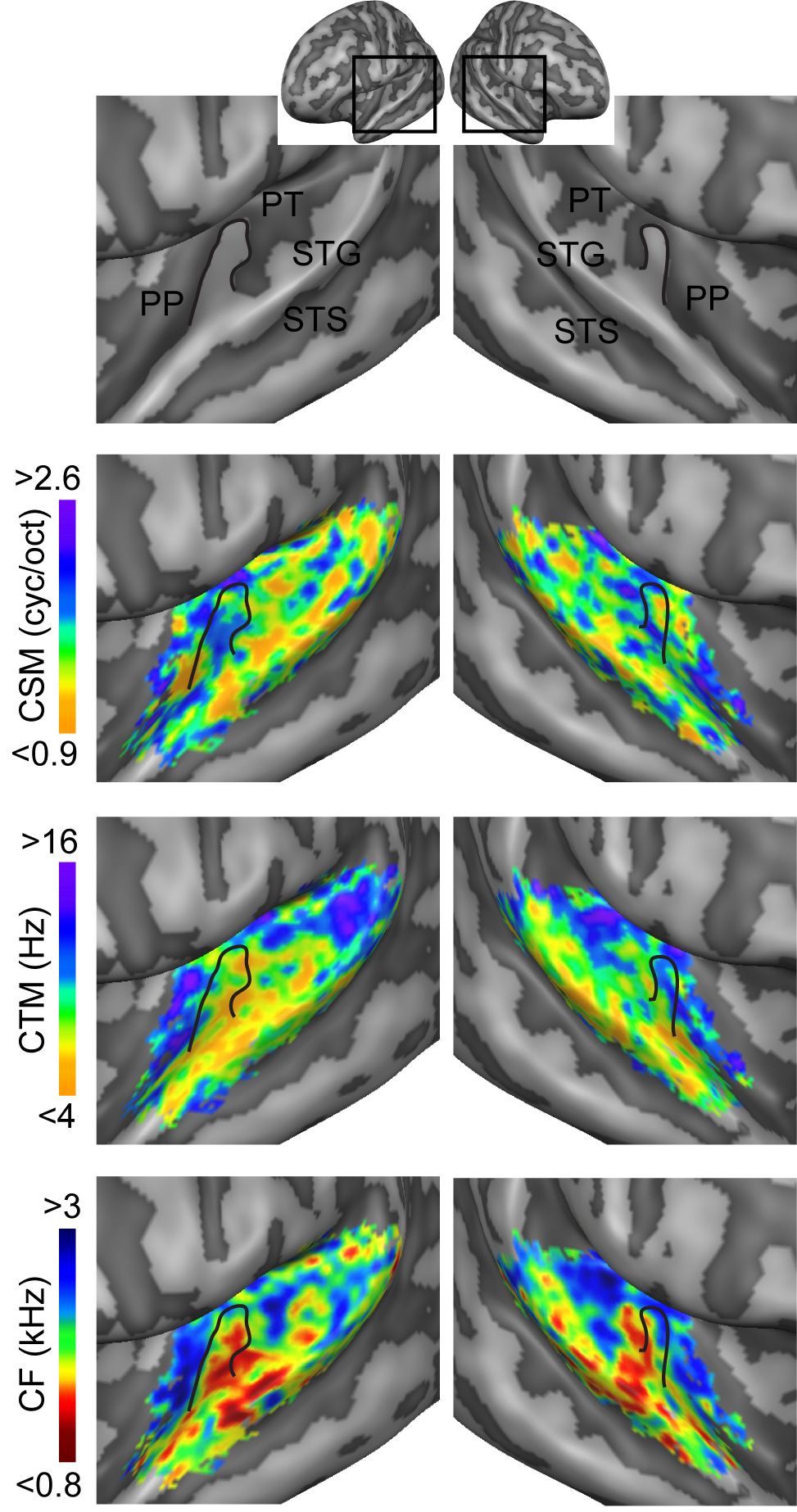

Supplement: Figure S8 — Unbiased topographic maps for the 7T dataset. Group maps of CSM, CTM and CF as derived from the joint frequency-specific MTF-based model while explicitly accounting for the effect of sound categories. The black line indicates HG. (TIF) [file pcbi.1003412.s008.tif]
