# Supplementary material for: Encoding of Natural Sounds at Multiple Spectral and Temporal Resolutions in the Human Auditory Cortex
Source: PLoS Comput Biol. 2014 Jan 2;10(1):e1003412. doi: 10.1371/journal.pcbi.1003412 (PMC3879146; doi:10.1371/journal.pcbi.1003412)
Supplement: Text S1 — Supplementary methods and references. (DOCX) [file pcbi.1003412.s009.docx]

**Text S1.** Supplementary Methods and References

**Supplementary methods**

*Subjects*

Five healthy, normal-hearing subjects (different for the two experiments; median age = 26 (32), 3 (3) males for the 3T (7T) experiment) gave their informed consent and participated in this study. Additionally to the main study, localizer data were collected in the same subjects for both the 3T and 7T experiment.

*Stimuli for the localizer experiment*

In the localizer experiment, we used Matlab (The MathWorks Inc.) to create amplitude modulated tones (8 Hz, modulation depth of 1) with a carrier frequency of 0.45, 0.5, and 0.55 kHz for the low-frequency condition; 1.35, 1.5, and 1.65 kHz for the middle frequency condition and 2.25, 2.5, and 2.75 kHz for the high-frequency condition. Sounds were sampled at 16 kHz and their duration was cut at 800 ms.

Sound onset and offset were ramped with a 10 ms linear slope, and their energy (RMS) levels were equalized. Before starting the measurement, sounds were played to the subject while headphones and earplugs were in place. Intensity of the sounds was further adjusted in order to equalize their perceived loudness.

*Magnetic Resonance Imaging*

In the 3T experiment, images were acquired on a 3T head only MR scanner (Siemens Allegra). Anatomical T1-weighted volumes covering the whole brain were obtained with an ADNI MPRAGE sequence (TR = 2250 ms; TE = 2.6 ms; matrix size = 256 x 256 x 192, voxel dimensions = 1 x 1 x 1 mm3). Functional T2*-weighted BOLD images were collected using a clustered volume EPI technique.

Data in the 7T experiment were acquired on a 7T whole body system driven by a Siemens console using a head gradient insert operating at up to 80 mT/m with a slew rate of 333 T/m/s. A head RF coil (single transmit, 16 receive channels) was used to acquire anatomical T1-weighted and functional T2*-weighted BOLD images. T1-weighted images were acquired using a modified MPRAGE sequence (TR=2500 ms; TI=1500 ms; TE=3.67 ms, voxel dimensions = 1 x 1 x 1 mm3). Proton density (PD) images were acquired together with the T1-weighted volumes and were used to minimize inhomogeneities in the T1-weighted images [1]. Acquisition time for anatomy was around 7 minutes. T2*-weighted functional data were acquired using a clustered volume EPI technique.

In the main experiment, fMRI time series were acquired according to a fast event-related scheme (3T experiment: TR = 2600 ms; time of acquisition [TA] = 1200 ms; TE = 30 ms; number of slices = 13; matrix size = 128 x 128; voxel size = 2 x 2 x 2 mm3, silent gap = 1400 ms; 7T experiment: TR = 2600 ms; TA = 1200 ms; TE = 30 ms; number of slices = 31; GRAPPA acceleration X3; partial Fourier 6/8; voxel size = 1.5 x 1.5 x 1.5 mm³, silent gap = 1400 ms).

The localizer was designed according to a blocked scheme. The acquisition parameters were (3T: TR = 3000 ms; TA = 1500 ms; TE = 30 ms; number of slices = 18; matrix size = 128 x 128; voxel size = 2 x 2 x 2 mm3, silent gap = 1500 ms; 7T: TR = 3000 ms; TA = 1500 ms; TE = 30 ms; number of slices = 44; GRAPPA acceleration X3; partial Fourier 6/8; voxel size = 1.5 x 1.5 x 1.5 mm3, silent gap = 1500 ms). Each block (~ 18 s) consisted of six sounds of the same condition (one sound per TR, presented in the silent gap). Per run, two blocks of each condition were presented (12 s of silence between blocks). Overall, the localizer consisted of six runs (~9 minutes each).

Analysis of anatomical and functional images was performed with BrainVoyager QX. Preprocessing consisted of slice scan-time correction (sinc interpolation), temporal high-pass filtering and 3-dimensional motion correction. Functional slices were co-registered to the anatomical data and normalized to Talairach space. In the 7T experiment, normalized functional data were resampled (sinc interpolation) to 1 mm isotropic. The border between gray and white matter was segmented from anatomical volumes and used to generate inflated hemispheres of the individual subjects. Finally, functional cortex based alignment [2] was performed to improve alignment across subjects and compute group maps.

*Reliability of parameters estimates: SNR computation*

Maps of voxels tuning preference were derived from the MTFs as estimated by the joint frequency-specific MTF-based model. To assess the reliability of the maps, we computed the signal-to-noise ratio (SNR) of the MTFs estimates, by applying a method developed to evaluate the reproducibility of spectro-temporal receptive fields (STRFs) as derived from neuronal responses [3,4]. We generated fifty bootstrap samples by drawing with repetition from the original set of responses, and voxels MTFs were estimated for each bootstrap sample. The same bootstrap samples were used for all voxels and subjects in order to prevent sample-related variability. The regularization parameter for the fitting procedure was constant across bootstraps and was set to the value derived when the model was trained on the original set of responses. For each voxel, the bootstrap procedure yielded a distribution of parameter estimates at each point *(ω,Ω,f)* of the MTF. The SNR at each *(ω,Ω,f)* was computed as the ratio between the average power and the variance across bootstraps, as follows:

$$SNR_{\omega,\Omega,f}=\frac{\left|\mu_{\omega,\Omega,f}\right|^{2}}{\sigma_{\omega,\Omega,f}^{2}}$$

where μ_ω,Ω,f_ is the mean across bootstraps.

The overall SNR of the MTF was then obtained as the power-weighted mean of all SNR_ω,Ω,f_ , where the power P_ω,Ω,f_ is the square of the MTF computed on the original set of responses (i.e. without bootstrap):

__

For every subject, cortical maps of SNR_MTF_ were generated by color-coding the computed SNR values and projecting them onto an inflated representation of the cortex (Figure S7). High SNR indicates reproducibility of MTF estimates across bootstraps.

*Computation of unbiased topographic maps*

To ensure that estimated maps of tuning preference were not confounded by the frequency and modulation content of sound categories, we recomputed maps of CF, CSM and CTM while explicitly accounting for confounding effects of sound category (this analysis was performed on the 7T dataset).

The sounds used in this experiment may be (arbitrarily) grouped into four categories:

1. HA: Human Animate (speech, voice)

2. NA: Nonhuman Animate (animal sounds)

3. HI: Human Inanimate (tools, music)

4. NI: Nonhuman Inanimate (sounds of nature)

For each category, we defined a categorical predictor that takes the value 1 if the stimulus belongs to that category and 0 otherwise. Next, we appended the categorical predictors to the joint frequency-specific MTF-based representation and estimated the parameters of the resulting model. Finally, we derived maps of voxels preferred spectral modulation, temporal modulation and frequency. The spatial patterns of the computed maps remained unchanged (Figure S8), indicating that voxels preferred features are estimated correctly despite differences in frequency and modulation content across sound categories.

**References**

1. Van de Moortele P-F, Auerbach EJ, Olman C, Yacoub E, Uğurbil K, et al. (2009) T1 weighted brain images at 7 Tesla unbiased for Proton Density, T2⁎ contrast and RF coil receive B1 sensitivity with simultaneous vessel visualization. NeuroImage 46: 432–446. Available: http://www.sciencedirect.com/science/article/pii/S1053811909001499.

2. Goebel R, Esposito F, Formisano E (2006) Analysis of functional image analysis contest (FIAC) data with brainvoyager QX: From single-subject to cortically aligned group general linear model analysis and self-organizing group independent component analysis. Hum Brain Mapp 27: 392–401. Available: http://dx.doi.org/10.1002/hbm.20249.

3. Shechter B, Depireux DA (2010) Nonlinearity of coding in primary auditory cortex of the awake ferret. Neuroscience 165: 612–620. Available: http://www.sciencedirect.com/science/article/pii/S0306452209017187.

4. Shechter B, Depireux DA (2012) Contrast tuned responses in primary auditory cortex of the awake ferret. European Journal of Neuroscience 35: 550–561. Available: http://dx.doi.org/10.1111/j.1460-9568.2011.07985.x.
